# Uncovering Mechanisms of *Zanthoxylum piperitum* Fruits for the Alleviation of Rheumatoid Arthritis Based on Network Pharmacology

**DOI:** 10.3390/biology10080703

**Published:** 2021-07-23

**Authors:** Kikwang Oh, Md. Adnan, Dongha Cho

**Affiliations:** Department of Bio-Health Convergence, College of Biomedical Science, Kangwon National University, Chuncheon 24341, Korea; nivirna07@kangwon.ac.kr (K.O.); mdadnan@kangwon.ac.kr (M.A.)

**Keywords:** *Zanthoxylum piperitum* fruits, rheumatoid arthritis, network pharmacology, MAPK signaling pathway, PPAR signaling pathway

## Abstract

**Simple Summary:**

The aim of the study is to investigate the bioactives of *Zanthoxylum piperitum* fruits on rheumatoid arthritis. The methodology to identify the relationship between signaling pathways, targets, and bioactives is based on network pharmacology. The results show that *Zanthoxylum piperitum* fruits might alleviate inflammatory symptoms of rheumatoid arthritis. Thus, we suggest that *Zanthoxylum piperitum* fruit is a promising herbal plant to reduce the level of cytokines against rheumatoid arthritis.

**Abstract:**

*Zanthoxylum piperitum* fruits (ZPFs) have been demonstrated favorable clinical efficacy on rheumatoid arthritis (RA), but its compounds and mechanisms against RA have not been elucidated. This study was to investigate the compounds and mechanisms of ZPFs to alleviate RA via network pharmacology. The compounds from ZPFs were detected by gas chromatography–mass spectrometry (GC-MS) and screened to select drug-likeness compounds through SwissADME. Targets associated with bioactive compounds or RA were identified utilizing bioinformatics databases. The signaling pathways related to RA were constructed; interactions among targets; and signaling pathways-targets-compounds (STC) were analyzed by RPackage. Finally, a molecular docking test (MDT) was performed to validate affinity between targets and compounds on key signaling pathway(s). GC-MS detected a total of 85 compounds from ZPFs, and drug-likeness properties accepted all compounds. A total of 216 targets associated with compounds 3377 RA targets and 101 targets between them were finally identified. Then, a bubble chart exhibited that inactivation of MAPK (mitogen-activated protein kinase) and activation of PPAR (peroxisome proliferator-activated receptor) signaling pathway might be key pathways against RA. Overall, this work suggests that seven compounds from ZPFs and eight targets might be multiple targets on RA and provide integrated pharmacological evidence to support the clinical efficacy of ZPFs on RA.

## 1. Introduction

Rheumatoid arthritis (RA) is a long-term systemic autoimmune disorder that deteriorates the synovial joints and is associated with gradual disability [1]. RA is a progressive inflammation caused by joint damage and its functional loss around the articular [2,3]. RA can present irrespective of age, diagnosed in around 1% of the population, brings huge social-economic burden [4]. The main factor causing RA is uncontrollable cytokine secretion due to bone damage; however, the etiology of RA is unknown [5,6]. Commonly, the anti-RA drugs administered are disease-modifying arthritis drugs (DMARDs) and non-steroidal anti-inflammatory drugs (NSAIDs) in most countries [7]. At present, prolonged administration of these drugs is involved in severe side effects such as upset stomach, nephrotoxicity, and electrolyte imbalance [8,9]. In contrast, an animal test demonstrated that the repeated dose treatment of plant leaf methanolic extraction with anti-RA efficacy did not alter liver and kidney function [10]. It implies that herbal medicine extracts against RA are better clinical safety than unnatural compounds. For a long time, herbal plants treating RA were essential resources due to their excellent clinical efficacy and low adverse effects [11].

*Zanthoxylum piperitum* (ZP) belongs to the Rutaceae family, which has been chiefly used as a condiment of food in Korea, Japan, and China [12]. It was reported that essential oil in ZP showed a 38% reduction of nitric oxide (NO) related to the occurrence and progression of inflammatory joint disease [13]. Another report demonstrated that the peel of ZP has potent anti-inflammatory activities by suppressing nuclear factor kappa-light-chain-enhancer of activated B cells (NF-κB) and caspase-1 activation in lipopolysaccharide (LPS)-induced RAW264.7 cells [14]. The studies give a hint that ZP might be a herbal medicine to alleviate RA. So far, active compounds and pharmacological mechanisms of ZPFs against RA have not been elucidated. Hence, studies of active compounds and mechanisms of ZPFs against RA should be investigated to prove their therapeutic value.

Network pharmacology is an integrated analytical methodology to understand multiple elements such as compounds, targets and pathways [15]. Network pharmacology can unravel the mechanism of compounds in herbal plants with an integrated concept [16]. Additionally, network pharmacology makes a point of “multiple-targets, multiple-compounds”, instead of “one-target, one-compound” [17]. Therefore, network pharmacology is an optimal method to explicate herbal plant issues. Currently, network pharmacology has been utilized to prove bioactive compounds and mechanisms of herbal plants against complex diseases [18,19].

**Figure 1:**
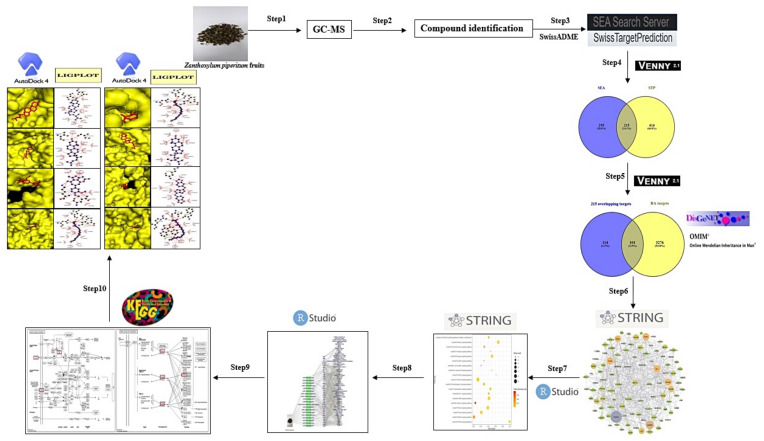
Workflow chart of network pharmacology analysis of ZPFs against RA.

Hence, network pharmacology was utilized to uncover the pharmacological mechanisms of bioactive compounds of ZPFs against RA. Firstly, compounds of ZPFs methanolic extraction identified from GC-MS filtered out drug-likeness candidates on a physicochemical descriptor tool. Secondly, targets related to filtered compounds were retrieved by public bioinformatic databases, and final overlapping targets calculated between compounds and RA targets retrieved by public disease target databases. Thirdly, a key target on protein–protein interaction (PPI) was identified, and two key signaling pathways of ZPFs against RA were identified by analyzing the final overlapping targets. Then, another key target of signaling pathways related to the occurrence and development of RA was identified by analyzing targets associated with signaling pathways. Finally, a molecular docking test (MDT) was carried out to find po from ZPFs against RA on each target related directly to two key signaling pathways. The workflow diagram is exhibited in Figure 1.

## 2. Materials and Methods

### 2.1. Plant Material Collection and Classification

The *Zanthoxylum piperitum* fruits (ZPFs) were collected from (latitude: 37.628975, longitude: 126.742978), Gyeonggi-do, Korea, in August 2020, and the plant was identified by Dr. Dong Ha Cho, Plant biologist and Professor, Department of Bio-Health Convergence, College of Biomedical Science, Kangwon National University. A voucher number (UUC 270) has been stored at Kenaf Corporation in the Department of Bio-Health Convergence, and the material can be used only for research purposes.

### 2.2. Plant Preparation, Extraction

The ZPFs were dried in a shady area at room temperature (20–22 °C) for 21 days, and dried ZPFs made powder using an electric blender. Approximately 50 g of ZPFs powder was soaked in 800 mL of 100% methanol (Daejung, Siheung city, Gyeonggi-do, Korea) for 10 days and repeated three times to achieve a high yield rate. The solvent extract was collected, filtered, and evaporated using a vacuum evaporator (IKA- RV8, Staufen city, Germany). The evaporated sample was dried under a boiling water bath (IKA-HB10, Staufen city, Germany) at 40 °C for around 8 h to obtain solid extraction.

### 2.3. GC-MS Analysis Condition

Agilent 7890A was used to carry out GC-MS analysis. GC was equipped with a DB-5 (30 m × 0.25 mm × 0.25 μm) capillary column. Initially, the instrument was maintained at a temperature of 100 °C for 2.1 min. The temperature was rose to 300 °C at the rate of 25 °C/min and maintained for 20 min. Injection port temperature and helium flow rate were ensured as 250 °C and 1.5 mL/min, respectively. The ionization voltage was 70 eV. The samples injected in split mode at 10:1. The MS scan range was set at 35–900 (*m/z*). The fragmentation patterns of mass spectra were compared with those stored in the using W8N05ST Library MS database (analyzed 28 April 2021). The percentage of each compound was calculated from the relative peak area of each compound in the chromatogram. The concept of integration was used in the ChemStation integrator algorithms (analyzed 28 April 2021) [20].

### 2.4. Chemical Compounds Identification and Drug-Likeness Screening

The chemical compounds from ZPFs were identified through GC-MS analysis. The compounds detected by GC-MS confirmed “drug-likeness” physicochemical property via Lipinski’s rule on SwissADME (http://www.swissadme.ch/) (accessed on 14 May 2021). The filtered compounds converted into SMILES (simplified molecular input line entry system) (accessed on 14 May 2021) format through PubChem (https://pubchem.ncbi.nlm.nih.gov/) (accessed on 14 May 2021).

### 2.5. Targets Associated with Compounds from ZPFs or Rheumatoid Arthritis

Targets related to the compounds were identified via both Similarity Ensemble Approach (SEA) (http://sea.bkslab.org/) (accessed on 16 May 2021) [21] and SwissTargetPrediction (STP) (http://www.swisstargetprediction.ch/) (accessed on 16 May 2021) [22] with “*Homo Sapiens*” mode, both of which is founded on SMILES (accessed on 14 May 2021). The RA-associated targets on humans were retrieved with DisGeNET (https://www.disgenet.org/) (accessed on 17 May 2021), OMIM (https://www.omim.org/) (accessed on 17 May 2021) and literature. The overlapping targets between compounds of ZPFs and RA-associated targets indicated by VENNY 2.1 (https://bioinfogp.cnb.csic.es/tools/venny/) (accessed on 18 May 2021).

### 2.6. PPI Networks and Bubble Chart

STRING (https://string-db.org/) (accessed on 19 May 2021) [23] was utilized to analyze the PPI network with final overlapping targets. The RPackage was utilized to identify the degree of value. Then, signaling pathways associated with the occurrence and development of RA were visualized on a bubble chart by RPackage; two key signaling pathways with the highest and the lowest rich factor were selected to analyze the relationships against RA.

### 2.7. Construction of STC Networks

The STC networks were utilized to construct a size plot based on the degree of values. In this size map, green rectangles (nodes) represented signaling pathways; gold triangles (nodes) stood for target proteins, and red circles (nodes) stood for compounds; its size represented degree value. The size of gold triangles represented the amount of connectivity with signaling pathways; the size of red circles represented the amount of connectivity with target proteins. The combined networks were constructed by using RPackage (analyzed 20 May 2021).

### 2.8. Preparation of Targets for MDT 

Firstly, two targets of MAPK signaling pathway, i.e., FGF2 (PDB ID: 1IIL), VEGFA (PDB ID: 3V2A), and one target of PPAR signaling pathway, i.e., PPARG (PDB ID: 3E00), were identified on STRING via RCSB PDB (https://www.rcsb.org/) (accessed on 21 May 2021). The final three targets selected as .pdb format were converted into .pdbqt format via Autodock (http://autodock.scripps.edu/) (accessed on 21 May 2021).

### 2.9. Preparation of Compounds from ZPFs for MDT

The ligand molecules were converted .sdf from PubChem into .pdb format using Pymol (accessed on 21 May 2021), and the ligand molecules were converted into .pdbqt format through Autodock (accessed on 21 May 2021).

### 2.10. Preparation of Positive Standard Ligands for MDT 

The number of two positive ligands on FGF2 antagonists, i.e., NSC172285 (PubChem ID: 299405), NSC37204 (PubChem ID: 235612), and the number of one positive ligand on VEGFA antagonist, i.e., BAW2881 (PubChem ID: 16004702), the number of three positive ligands on PPARG antagonists, i.e., Pioglitazone (PubChem ID: 4829), Rosiglitazone (PubChem ID: 77999), Lobeglitazone (PubChem ID: 9826451) were selected to verify the docking score.

### 2.11. Ligand-Protein Docking

The ligand molecules were docked with target proteins utilizing autodock4 by setting up four energy ranges and eight exhaustiveness as default to obtain 10 different poses of ligand molecules [24]. The 2D binding interactions were used with LigPlot+ v.2.2 (https://www.ebi.ac.uk/thornton-srv/software/LigPlus/) (accessed on 21 May 2021). After docking, ligands of the lowest binding energy (highest affinity) were selected to visualize the ligand–protein interaction in Pymol (Schrödinger, New York, NY, USA). 

### 2.12. Toxicological Properties Prediction by AdmetSAR 

Toxicological properties of the key bioactive were established using the admetSAR web-service tool (http://lmmd.ecust.edu.cn/admetsar1/predict/) (accessed on 23 May 2021) because toxicity is an essential factor to develop new drugs. Hence, Ames toxicity, carcinogenic properties, acute oral toxicity, and rat acute toxicity were predicted by admetSAR (East China University of Science and Technology, Shanghai, China).

## 3. Results

### 3.1. Chemical Compounds from ZPFs

**Figure 2:**
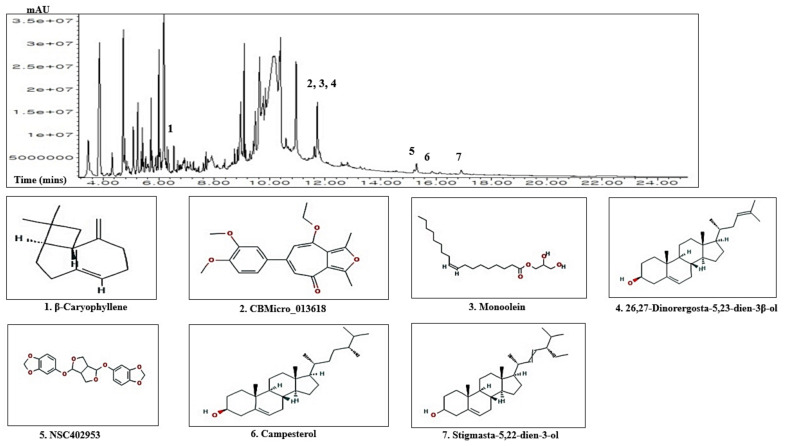
A typical GC-MS peak of ZPFs methanolic extract and the number of seven key compounds.

A total of 85 chemical compounds and its seven key chemical compounds in ZPFs were detected by the GC-MS analysis (Figure 2), and the name of compounds, PubChem ID, retention time (mins), and peak area (%) were enlisted in Table 1. Lipinski’s rules accepted all 85 compounds (molecular weight ≤500g/mol; Moriguchi octanol-water partition coefficient ≤4.15; number of nitrogen or oxygen ≤10; number of NH or OH ≤5), and all chemical compounds satisfied with the criteria of “Abbott Bioavailability Score (>0.1)” through SwissADME. The TPSA (topological polar surface area) value of chemical compounds was also accepted (Table 2).

### 3.2. Overlapping Targets between SEA and STP Related to Chemical Compounds

**Figure 3:**
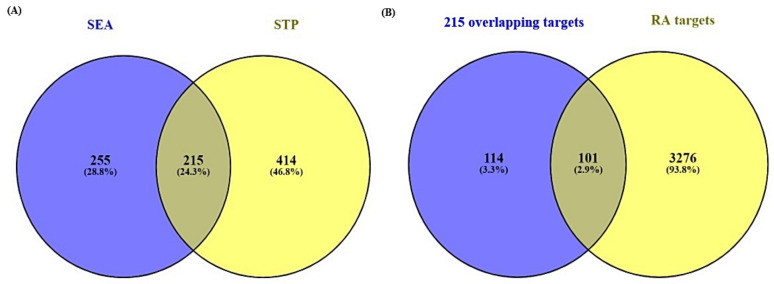
(**A**) The number of 215 overlapping targets between SEA (470 targets) and STP (629 targets). (**B**) The number of 101 final overlapping targets between 215 overlapping targets from two databases (SEA and STP) and RA associated with targets (3377 targets).

A total of 470 targets from Similarity Ensemble Approach (SEA) and 629 targets from SwissTargetPrediction (STP) were associated with 85 chemical compounds (Supplementary Table S1). The Venn diagram showed that 215 targets overlapped between the two public databases (Supplementary Table S1) (Figure 3A).

### 3.3. Overlapping Targets between RA-Related Genes and the Final 101 Overlapping Targets

A total of 3377 targets associated with RA were selected by retrieving from DisGeNET, Online Mendelian Inheritance in Man (OMIM) databases and literature (Supplementary Table S2). Venn diagram’s result displayed 101 overlapping targets that were selected between 3377 targets related to RA and the 215 overlapping targets (Figure 3B) (Supplementary Table S3).

### 3.4. Acquisition of a Key Target from PPI Networks

**Figure 4:**
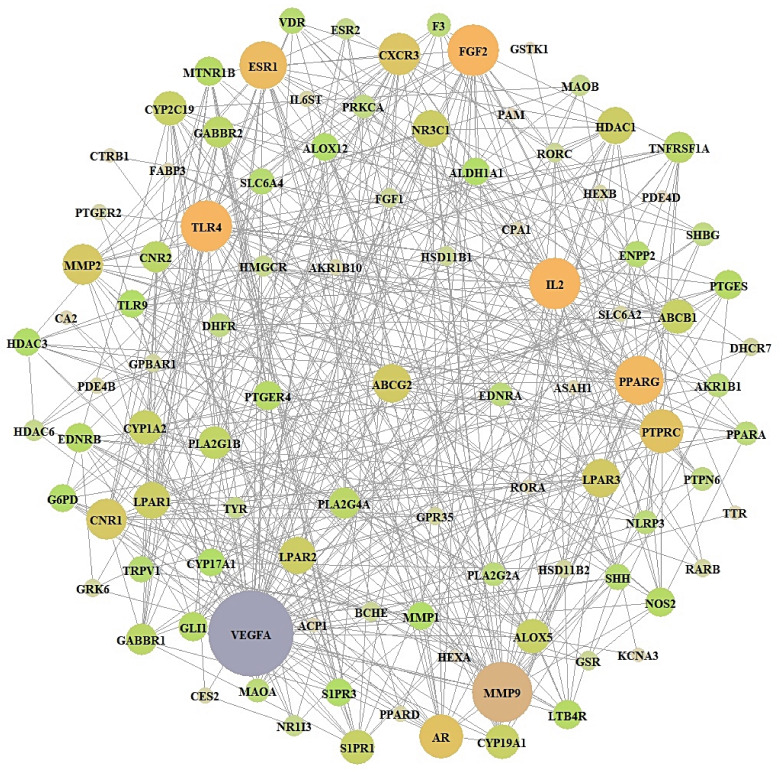
PPI networks (99 nodes, 469 edges). The size of the circle: degree of values.

From STRING analysis, 99 out of 101 overlapping targets were directly associated with RA occurrence and development, indicating 99 nodes and 469 edges (Figure 4). The two removed targets (CA1 and CA3) had no connectivity to the overlapping 101 targets. In PPI networks, Vascular Endothelial Growth Factor A (VEGFA) was the highest degree (42) and is considered a key target (Table 3).

### 3.5. Identification of Two Key Signaling Pathways from a Bubble Chart

**Figure 5:**
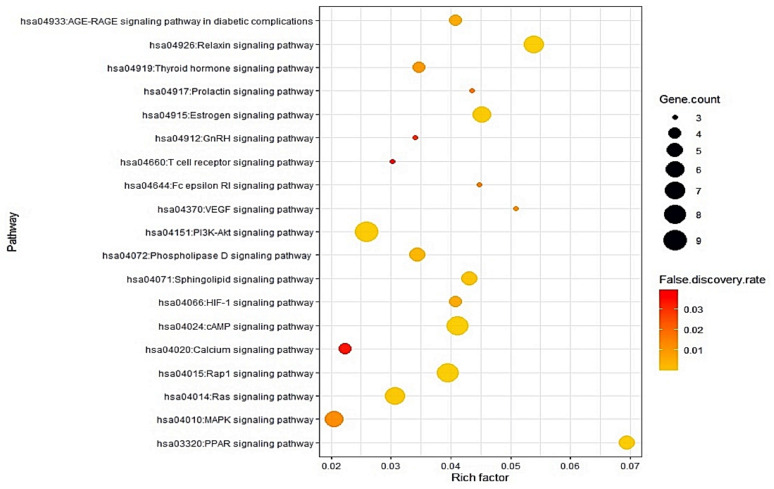
Bubble chart of 19 signaling pathways associated with cancer.

The results of Kyoto Encyclopedia of Genes and Genomes (KEGG) pathway enrichment analysis unveiled that 19 signaling pathways were connected with 40 out of 101 targets (false discovery rate <0.05). The 19 signaling pathways were directly related to RA, indicating that these 19 signaling pathways might be significant pathways of ZPFs against RA. The description of 19 signaling pathways were shown in Table 4. Additionally, a bubble chart indicated that inactivation of MAPK signaling pathway and activation of PPAR signaling pathway might be key signaling pathways of ZPFs to alleviate RA (Figure 5). Specifically, MAPK signaling pathway is associated with VEGFA (a hub target) in holistic PPI networks while PPAR signaling pathway is not related to VEGFA.

### 3.6. Construction of Signaling Pathway-Target-Compound Network

**Figure 6:**
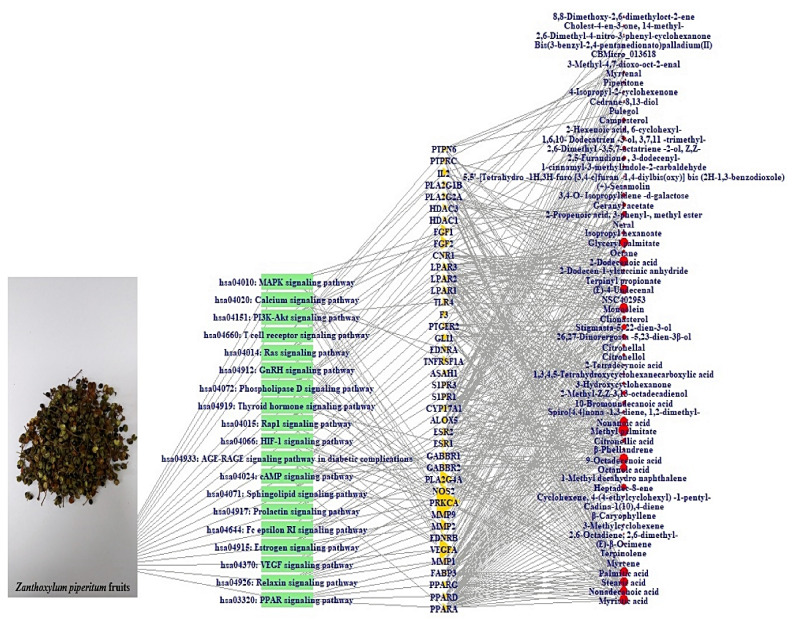
STC networks (122 nodes, 488 edges). Green rectangle: signaling pathway; gold triangle: targets; red circle: compounds.

The signaling pathway–target–compound (STC) network is displayed in Figure 6. There were 19 pathways, 40 targets, and 63 compounds (122 nodes, 488 edges). The nodes stood for the total number of relationships between signaling pathways, targets, and compounds. The edges represented the relationship of three elements (19 pathways, 40 targets, and 63 compounds). The STC network suggested that the uppermost target is Protein Kinase C Alpha (PRKCA) with 14 degrees among 19 signaling pathways (Table 5).

### 3.7. KEGG Pathway Enrichment Analysis

**Figure 7:**
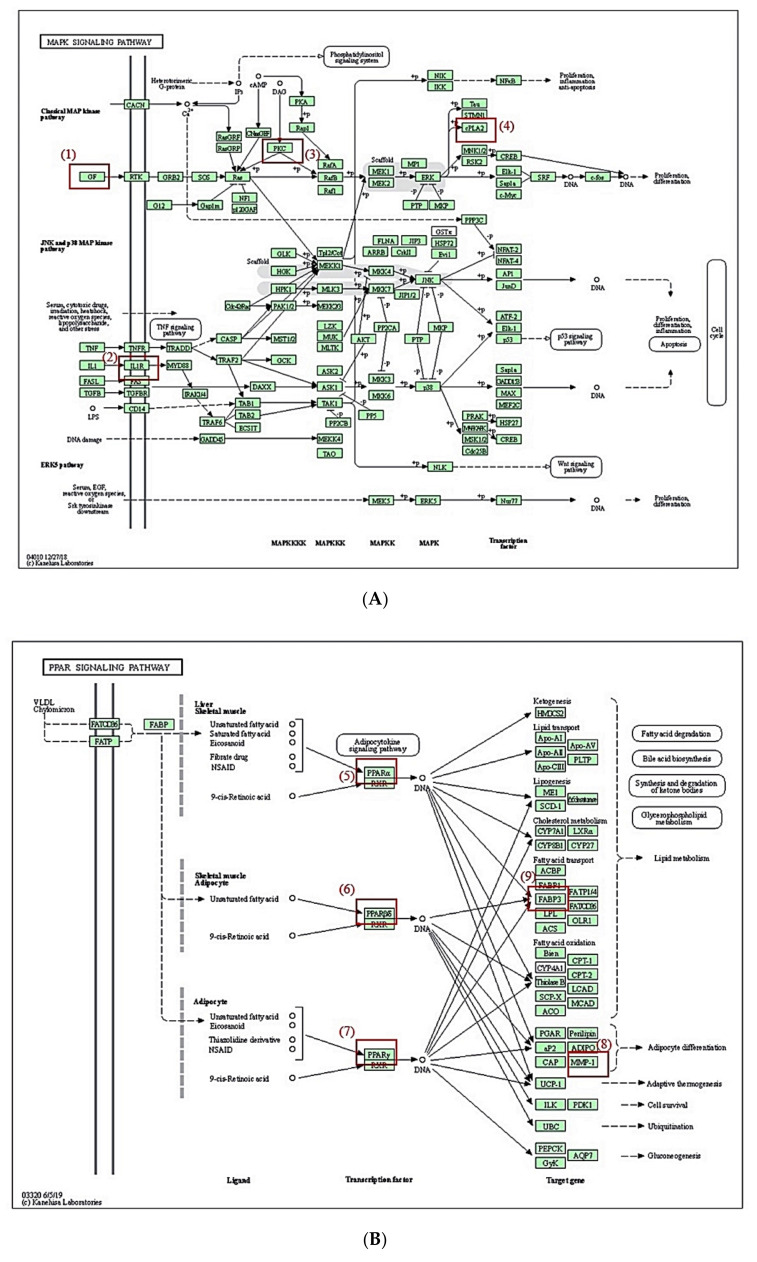
KEGG pathway enrichment map. (**A**) MAPK signaling pathway. (**B**) PPAR signaling pathway. Red rectangles represent key targets: (1) FGF1, FGF2, VEGFA; (2) TNFRSF1A; (3) PRKCA; (4) PLA2G4A; (5) PPARA; (6) PPARD; (7) PPARG; (8) MMP1; (9) FABP3.

KEGG pathway enrichment analysis [25] reveals that 19 signaling pathways are associated with the occurrence and development of RA. Out of 19 signaling pathways, MAPK signaling pathway and PPAR signaling pathway were most significantly related to RA (Figure 7). The red rectangles indicated core targets on the two signaling pathways. One of the MAPK signaling pathways, PRKCA (a species of PKC), is an excellent immunomodulatory target, which is linked to the onset of inflammatory arthritis, including RA [26]. It was subsequently shown that inactivation of PRKCA results in the inhibition of c-fos; eventually, the cascade leads to the amelioration of RA in an animal model [27]. One of the PPAR signaling pathways, FABP3, is located in the upstream region to regulate lipid metabolism. It was reported that defective lipid metabolism was observed in RA patients who are persistent to proinflammatory responses [28]. 

### 3.8. MDT of 6 Targets and 23 Chemical Compounds Associated with MAPK Signaling Pathway

From both SEA and STP databases, it was uncovered that FGF1 is associated with two chemical compounds, FGF2 with four chemical compounds, VEGFA with five chemical compounds, TNFRSF1A with two chemical compounds, PRKCA with 16 chemical compounds, and PLA2G4A with four chemical compounds. Out of 33 chemical compounds, 10 chemical compounds overlapped, and finally, 23 chemical compounds were identified on the MAPK signaling pathway.

**Figure 8:**
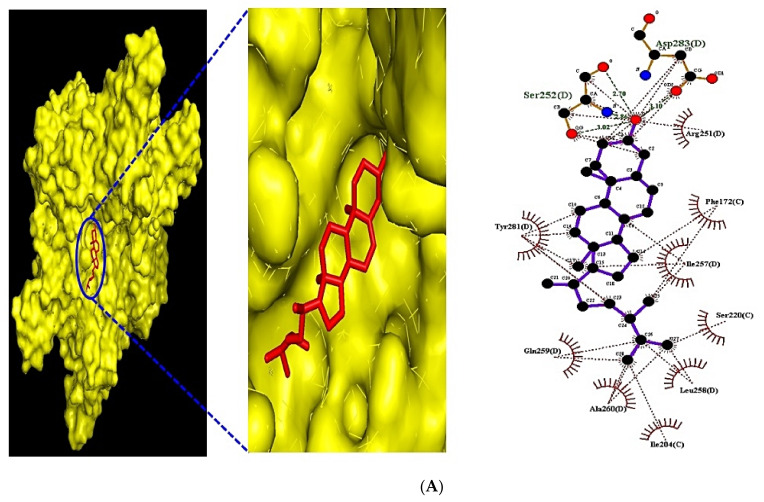

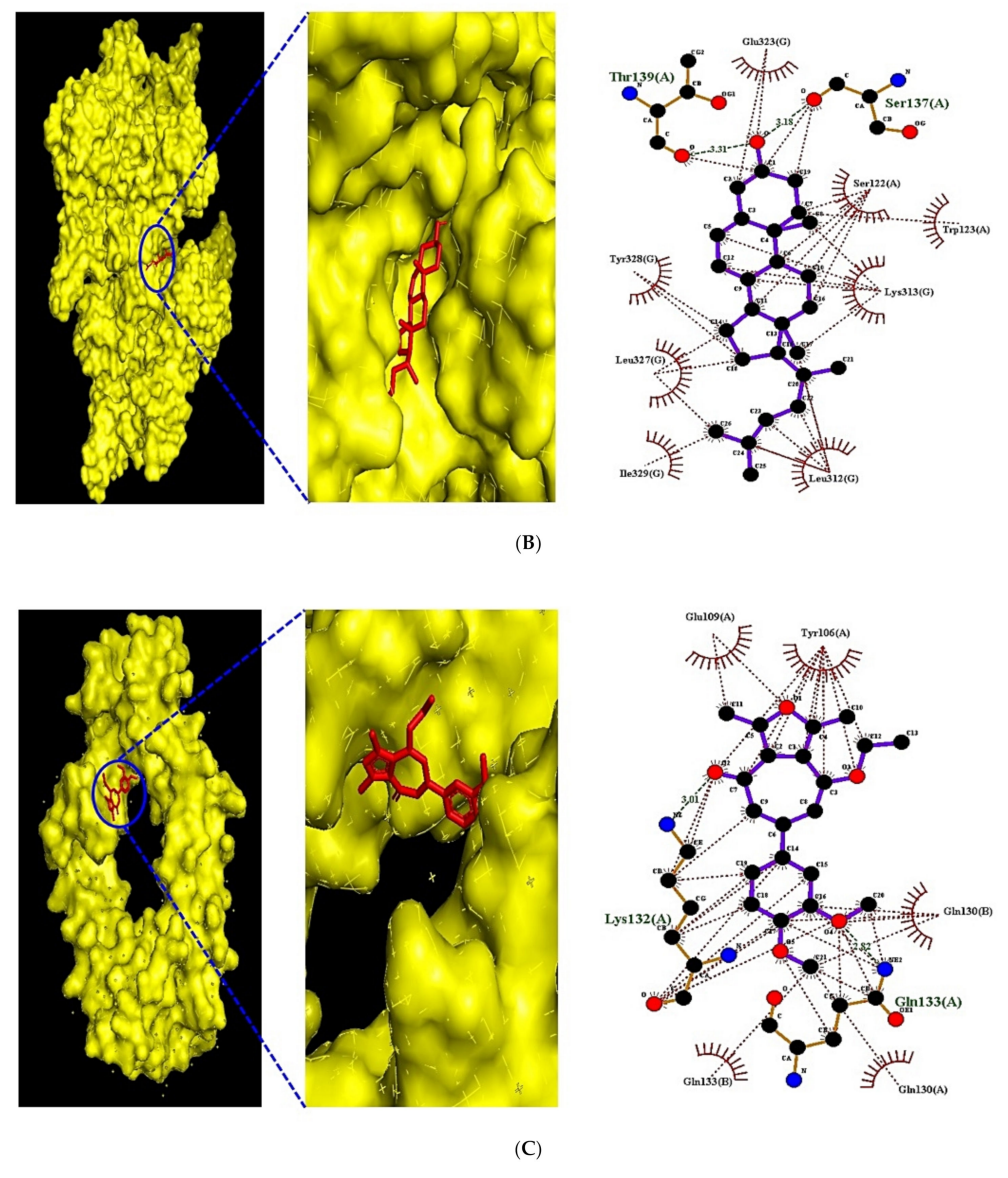

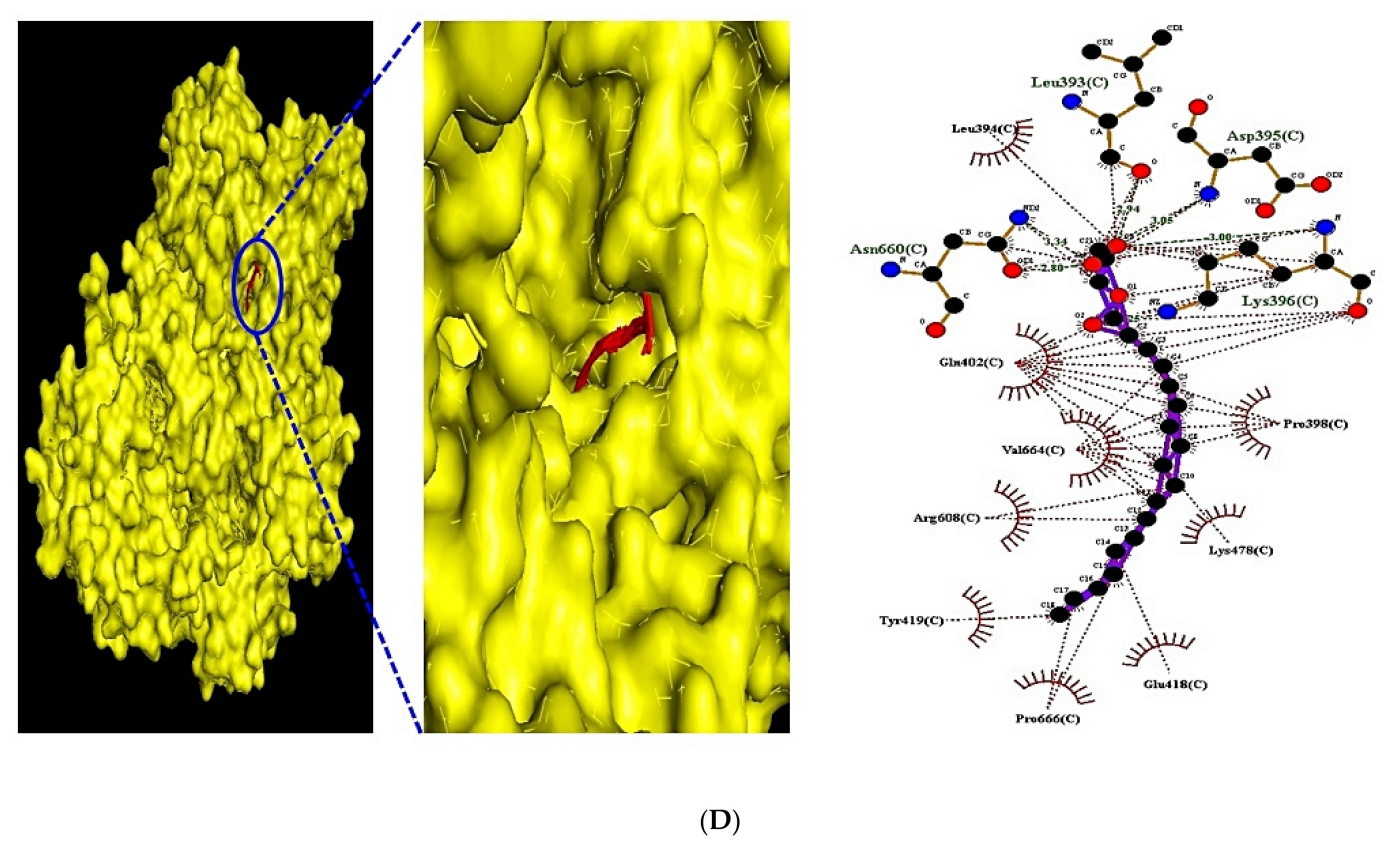
(**A**) MDT of campesterol (PubChem ID: 173183) on FGF1 (PDB ID: 3OJ2). (**B**) MDT of 26,27-Dinorergosta-5,23-dien-3β-ol (PubChem ID: 22213488) on FGF2 (PDB ID: 1IIL). (**C**) MDT of CBMicro_013618 (PubChem ID: 1109374) on TNFRSF1A (PDB ID: 1NCF). (**D**) MDT of monoolein (PubChem ID: 5283468) on PRKCA (PDB ID: 1NCF).

The MDT performed to evaluate affinity between target and ligand displayed the greatest affinity complex, depicted in Figure 8. In detail, campesterol (−8.4 kcal/mol) docked on the FGF1 target (PDB ID: 3OJ2) had the greatest affinity; however, it had a lower affinity than suramin sodium (−19.1 kcal) as a positive control. The 26,27-Dinorergosta-5,23-dien-3β-ol (−8.0 kcal/mol) docked on FGF2 target (PDB ID: 1IIL) had the highest affinity; its affinity was lower than NSC172285 (−14.7 kcal/mol) as a positive control. The 3,4-O-Isopropylidene-d-galactose (−5.3 kcal/mol) docked on VEGFA (PDB ID: 3V2A) had the highest affinity; however, the affinity score was invalid (> −6.0 kcal/mol) [29]. The CBMicro_013618 docked on TNFRSF1A (PDB ID: INCF) had the greatest affinity. Moreover, its score was better than Enamin_004209 (−5.3 kcal/mol) as a positive control. The berberine (−6.6 kcal/mol) as a positive control had a higher affinity than the stearic acid (−4.5 kcal/mol) docked on PLA2G4A (PDB ID: 1BCI). The monoolein (−6.7 kcal/mol) had the greatest affinity on PRKCA (PDB ID: 3IW4). Furthermore, its affinity score was greater than sphingosine (−5.5 kcal/mol) as a positive control. The detailed docking information is listed in Table 6.

### 3.9. MDT of 5 Targets and 43 Chemical Compounds Associated with PPAR Signaling Pathway

**Figure 9:**
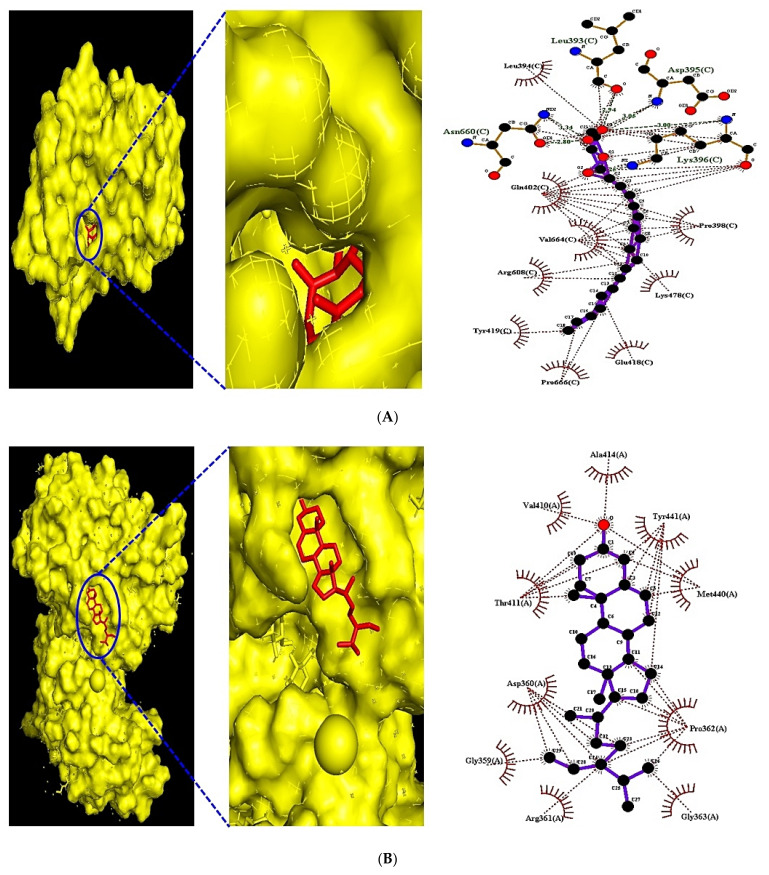

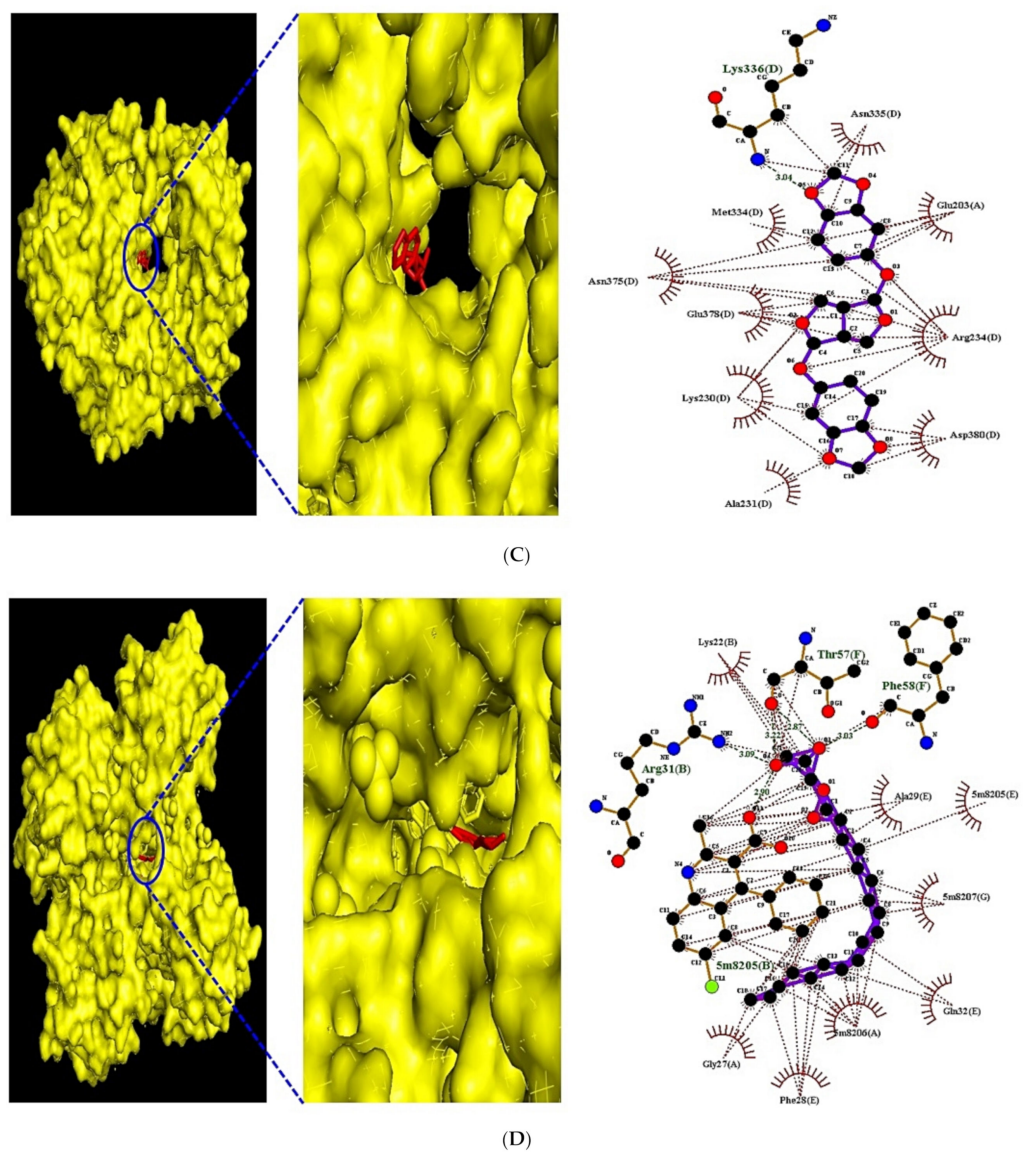
(**A**) MDT of β-Caryophyllene (PubChem ID: 5281515) on PPARA (PDB ID: 3SP6). (**B**) MDT of Stigmasta-5,22-dien-3-ol (PubChem ID: 53870683) on PPARD (PDB ID: 5U3Q). (**C**) MDT of NSC402953 (PubChem ID:345349) on PPARG (PDB ID: 3E00). (**D**) MDT of monoolein (PubChem ID: 5283468) on FABP3 (PDB ID: 5HZ9).

From both SEA and STP databases, it was revealed that Peroxisome Proliferator-Activated Receptor Alpha (PPARA) is related to 32 chemical compounds, Peroxisome Proliferator-Activated Receptor Delta (PPARD) to 18 chemical compounds, Peroxisome Proliferator-Activated Receptor Gamma (PPARG) to 17 chemical compounds, FABP3 to 26 chemical compounds, and MMP to five chemical compounds. The MDT performed to obtain affinity between targets and ligands exhibited the highest affinity complex, as displayed in Figure 9. Noticeably, β-Caryophyllene (−8.6 kcal/mol) docked on PPARA (PDB ID: 3SP6) had the greatest affinity, which had a higher score than Clofibrate (−6.4 kcal/mol), Gemfibrozil (−6.3 kcal/mol), Ciprofibrate (−5.4 kcal/mol), Bezafibrate (−5.8 kcal/mol), and Fenofibrate (−5.4 kcal/mol) as five positive controls. Stigmasta-5,22-dien-3-ol (−8.6 kcal/mol) docked on PPARD (PDB ID: 5U3Q) had the greatest affinity, which had a better score than Cardarine (−8.5 kcal/mol) as a positive control. NSC402953 (−8.2 kcal/mol) docked on PPARG (PDB ID: 3E00) had the highest affinity, which had better than Pioglitazone (−7.7 kcal/mol), Rosiglitazone (−7.4 kcal/mol), and Lobeglitazone (−7.3 kcal/mol) as three positive controls. Monoolein (−8.9 kcal/mol) docked on FABP3 (PDB ID: 5HZ9) had the greatest affinity; specifically, there was no positive control on FABP3 (PDB ID: 5HZ9). 2-Propenoic acid, 3-phenyl-, methyl ester (−5.0 kcal/mol) docked on MMP1 (PDB ID: 1SU3) had the highest affinity, which had lower affinity than Batimastat (−6.7 kcal/mol), and Ilomastat (−6.5 kcal/mol) as two positive controls. The detailed docking information is listed in Table 7.

### 3.10. Identification of the Uppermost Seven Targets and Eight Compounds from Two Key Signaling Pathways against RA

Campesterol on FGF1, 26,27-Dinorergosta-5,23-dien-3β-ol on FGF2, CBMicro_013618 on TNFRSF1A, and monoolein on PRKCA on MAPK signaling pathway had significant valid affinity score to develop new promising ligands. Additionally, β-Caryophyllene on PPARA, Stigmasta-5,22-dien-3-ol on PPARD, NSC0402953 on PPARG, and monoolein on FABP3 had an important valid score on the PPAR signaling pathway.

### 3.11. Toxicological Properties of 8 Compounds

Additionally, toxicological properties of Campesterol; 26,27-Dinorergosta-5,23-dien-3β-ol; CBMicro_013618; monoolein; β-Caryophyllene; Stigmasta-5,22-dien-3-ol; and NSC0402953 were predicted by admetSAR online tool. Our result indicated that chemical compounds did not disclose Ames toxicity, carcinogenic properties, acute oral toxicity, and rat acute toxicity properties (Table 8).

## 4. Discussion

PPI network showed that the therapeutic efficacy of ZPFs on RA was associated with 99 targets. The KEGG pathway analysis enrichment of 99 targets revealed that 19 signaling pathways were related closely to the occurrence and progression of RA, suggesting that these signaling pathways might be remedial mechanisms of ZPFs to alleviate RA. The relationships of the 19 signaling pathways with RA are briefly discussed as follows. PPAR signaling pathway: the activation of PPAR is a good strategy to alleviate RA, which can suppress the inflammatory activity of NF-κB in fibroblast-like synoviocytes (FLSs) [30]. Relaxin signaling pathway: the combined relaxin with estrogen exerts anti-inflammatory effects by dampening neutrophil function [31]. Vascular Endothelial Growth Factor (VEGF) signaling pathway: VEGF expression level in RA patients increased significantly, compared with healthy groups; moreover, patients under RA for an extended period exerted higher VEGF expression level in serum [32]. Estrogen signaling pathway: the estrogen treatment might have an inhibitory effect on RA symptoms or delay in the onset of disease, and estrogen has anti-inflammatory activity in an animal test of RA [33]. Fc epsilon RI signaling pathway: Fc epsilon RI signaling pathway is associated with inflammatory etiology, antigen-induced autoimmune reaction, and control of lipid metabolic pathway, and damage in human joint [34]. Prolactin signaling pathway: Prolactin collaborates with other proinflammatory factors to stimulate macrophages through prolactin receptors which might be a potential therapeutic target in RA [35]. Sphingolipid signaling pathway: Sphingolipid expression level in RA elevated in the serum sample, consistent with known roles of sphingolipids related to inflammation [36]. Cyclic AMP (cAMP) signaling pathway: stimuli-induced cAMP production can exert proinflammatory effects in RA [37]. It implies that the activation of the cAMP level might drive inflammatory responses. AGE-RAGE (the receptor for advanced glycation end products) signaling pathway in diabetic complications: the expression of RAGE increased in RA patients, and IL-17 and IL-1β triggered RAGE production [37]. It suggests that the inhibition of RAGE might be a good target for RA treatment. Hypoxia-Inducible Factor-1(HIF-1) signaling pathway: synovial hypoxia is characterized by RA patients, leading to inflammation, cartilage destruction, and oxidative impairment [38]. Ras-associated protein-1 (RAP1) signaling pathway: The downregulation of RAP1 in RA induces ongoing inflammation due to the overproduction of free radicals [39]. Thyroid hormone signaling pathway: joint damages in thyroid gland abnormalities are due to hypothyroidism, and thyroid hormone plays a vital role in antioxidant modulation, as indicated by in vivo and in vitro tests [40,41]. Phospholipase D signaling pathway: phospholipase D1 (PLD1) is a mediator to induce proinflammatory cytokines with a reduction of the regulatory T (Treg) cell and recruitment of Th17 cell in collagen-induced arthritis mice [42]. Gonadotropin-releasing hormone (GnRH) signaling pathway: GnRH is a potent substance to alleviate inflammation in RA patients with a high level of GnRH [43]. Ras signaling pathway: T cells in RA patients show the Ras signalling pathway’s overactivation-related deeply to inflammation [44]. T cell receptor signaling pathway: T cell recruitment to the inflammatory sites can induce chronic inflammation and autoimmunity [45]. Phosphoinositide 3-kinase—Akt (PI3K-Akt) signaling pathway: a report demonstrated that PI3K-Akt signaling pathway was excessively activated, aggravating in overexpression Bcl-2, Mcl-1, and FLIP to result in unbalanced apoptosis of synovial cells, which is associated with occurrence and progression of RA [46,47]. Calcium signaling pathway: it was reported that the cytoplasmic calcium concentration in RA naïve CD4^+^ T cells is increased significantly [48]. It implies that the calcium signaling pathway is involved intensely in inflammatory responses.

MAPK signaling pathway: a study demonstrated that andrographolide with anti-inflammatory activities has potent anti-RA efficacy, inhibiting MAPK pathway [49]. This report is consistent with our result via network pharmacology analysis.

**Figure 10:**
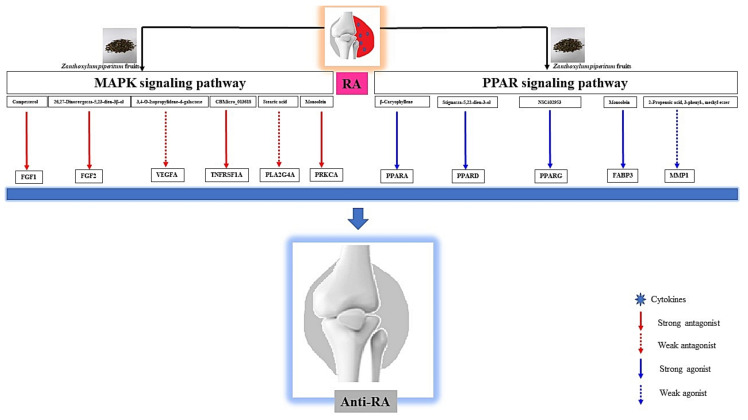
Schematic representation of key findings in the study.

To sum things up, the relationship between compounds and targets through the network pharmacology concept was clarified, and ligands with the most excellent affinity out of selective ligands in ZPFs were considered as potential therapeutic candidates against RA, compared with existing ligands via MDT. The PPI network suggested that VEGFA is the highest degree of value (42) on the MAPK signaling pathway. However, the affinity of ZPFs compounds on VEGFA was invalid (> −6.0kcal/mol) [29]; furthermore, existing ligand (BAW2881: −7.6 kcal/mol) had better affinity than a compound (3,4-O-Isopropylidene-d-galactose: −5.3 kcal/mol) with the greatest affinity from ZPFs. The STC network indicated that PRKCA had the greatest degree of value (14) on the MAPK signaling pathway, and monoolein (PubChem ID: 5283468) had the greatest affinity (−6.7 kcal/mol), which was superior to an existing ligand (sphingosine: −5.5 kcal/mol). On PPAR signaling pathway, β-Caryophyllene (−8.6 kcal/mol) on PPARA, Stigmasta-5,22-dien-3-ol (−8.6 kcal/mol) on PPARD, and NSC402953 (−8.2 kcal/mol) on PPARG had better affinity than existing ligands on each target. Specifically, monoolein on FABP3 had the greatest affinity (−8.9 kcal/mol). Moreover, agonists of FABP3 were not reported yet. Furthermore, there were no valid ligands on MMP1 on the PPAR signaling pathway. Taken together, the most noticeable ligand of ZPFs against RA is monoolein with dual effects on RA. Monoolein might be an antagonist on the MAPK signaling pathway or an agonist on the PPAR signaling pathway (Figure 10). A study demonstrated that monoolein treatment in LPS- stimulated primary murine bone marrow-derived dendritic cells (BMDCs) inhibited the activation of MAPK and NF-κB; moreover, it suppressed the production of NO and iNOS in RAW264.7 cells [50]. This suggests that the inactivation of the MAPK signaling pathway is a good strategy to ameliorate inflammation associated with bone damage. Another report indicated that the expression level of PPARG in FLSs of RA patients was significantly reduced compared with healthy FLSs [30]; this implies that the activation of the PPAR signaling pathway might be a therapeutic mechanism against RA. These reports are in line with our results. Additionally, we suggested that monoolein might be an antagonist in activating PRKCA on MAPK signaling pathway and might also be an agonist to activate FABP3 on the PPAR signaling pathway.

## 5. Conclusions

This study provides eight targets, seven compounds, and two key signaling pathways of ZPFs which show promise against RA, which might be useful for multiple target combination therapies on RA. Out of seven compounds from ZPFs, monoolein shows dual effects: inactivation of MAPK signaling pathway and activation of the PPAR signaling pathway. The key pharmacological mechanisms of ZPFs against RA might be to inhibit cytokine production in synovial cells by binding on PRKCA or FABP3.

## Figures and Tables

**Table 1:** A list of 85 chemical compounds identified from ZPFs via GC-MS and profiling of bioactivities.

No.	Compounds	Pubchem ID	RT (mins)	Area (%)	Pharmacological Activities (Reference)
1	Myrcene	31253	3.462	1.49	Antibacterial, Antioxidant, Fungicide
2	3(5)-[[1,2-Dihydroxy-3-propoxy]methyl]-4-hydroxy-1H-pyrazole-5(3)-carboxamide	135747301	3.683	0.09	No reported
3	β-Phellandrene	11142	3.866	4.28	Fungicide
4	Hex-3-yne	13568	3.971	0.10	No reported
5	3-Hydroxycyclohexanone	439950	4.087	0.06	No reported
6	Isopropyl hexanoate	16832	4.145	0.12	No reported
7	Terpinolene	11463	4.250, 4.318	0.59	Fungicide, Antioxidant
8	Vinylcyclooctane	93331	4.520	0.07	No reported
9	2-Tetradecynoic acid	324386	4.587	0.10	No reported
10	Citronellal	7794	4.721	2.75	Antibacterial, Fungicide
11	3-Hydroxy-2,3-dihydromaltol	119838	4.779	0.67	No reported
12	Pulegol	92793	4.856	0.29	No reported
13	Octanoic acid	379	4.923	0.24	Candidicide, Fungicide
14	(E)-4-Undecenal	5283357	4.981	0.10	No reported
15	4-Isopropyl-2-cyclohexenone	92780	5.087	1.04	No reported
16	Citronellol	8842	5.241	1.20	Antibacterial, Candidicide, Sedative
17	(E)-beta-Ocimene	5281553	5.366	0.39	Insecticide
18	3,7-Dimethylocta-2,6-dien-1-ol	4458	5.414	0.65	No reported
19	Spiro[4 .4]nona-1,3-diene, 1,2-dimethyl-	570800	5.452	0.21	No reported
20	Piperitone	6987	5.529	0.41	Antiasthmatic
21	Nonanoic acid	8158	5.606	0.42	Perfumery
22	8,8-Dimethoxy-2,6-dimethyloct-2-ene	102507	5.721	0.98	No reported
23	p-Isopropylbenzyl formate	105515	5.760	0.40	No reported
24	Citronellic acid	10402	5.895	0.55	No reported
25	α-Terpinene	7462	5.952	0.24	Antispasmodic
26	2,6-Octadiene, 2,6-dimethyl-	5365898	6.000	1.60	No reported
27	Terpinyl propionate	62328	6.048	0.43	No reported
28	Geranyl acetate	1549026	6.193	4.60	Sedative
29	3-Methylcyclohexene	11573	6.250	0.21	No reported
30	1,4-Dimethyl-4beta-methoxy-2,5-cyclohexadien-1α-ol	12561656	6.298	0.33	No reported
31	2-Propenoic acid, 3-phenyl-, methyl ester	7644	6.346	0.49	No reported
32	6-Methylenespiro[4.5]decane	564762	6.471	0.07	No reported
33	β-Caryophyllene	5281515	6.539	0.67	Antibacterial, Antiinflammation
34	Bergamotane	86000267	6.625	0.09	No reported
35	3-Methyl-4,7-dioxo-oct-2-enal	5363705	6.693	0.30	No reported
36	2,6-Dimethyl-3,5,7-octatriene-2-ol, Z,Z-	5363692	6.779	0.24	No reported
37	2-Dodecenoic acid	5282729	6.818	0.12	No reported
38	1,6,10-Dodecatrien-3-ol, 3,7,11-trimethyl-	8888	6.885	0.35	No reported
39	1-Methyldecahydronaphthalene	34193	6.943	0.46	No reported
40	Cadina-1(10),4-diene	10223	7.029	0.34	No reported
41	2-(4-Methylcyclohexyl)prop-2-en-1-ol	543946	7.135	0.42	No reported
42	Tetradec-13-enal	522841	7.250	0.24	No reported
43	9-Octadecenoic acid	965	7.308	0.15	Antiinflammation, Antileukotriene
44	1,2-Di-but-2-enyl-cyclohexane	5367574	7.375	0.10	No reported
45	4,12,12-trimethyl-9-methylene-5-oxatricyclo[8.2.0.04,6]dodecane	73555586	7.433	0.11	No reported
46	3,4-O-Isopropylidene-d-galactose	54504880	7.568	0.08	No reported
47	2-Hexenoic acid, 6-cyclohexyl-	5367614	7.616	0.22	No reported
48	Heptadec-8-ene	520230	7.693	0.35	No reported
49	Octane	356	7.779	0.35	No reported
50	Myristic acid	11005	7.827, 8.096	0.44	Anticancer, Antioxidant
51	D-(-)-Kinic Acid	1064	7.914	1.35	No reported
52	Nonadecanoic acid	12591	8.135	0.35	No reported
53	10-Bromoundecanoic acid	543401	8.337, 8.385	0.77	No reported
54	Stearic acid	5281	8.520	0.23	Hypocholesterolemic
55	Cysteamine S-sulfate	76242	8.587, 9.231, 9.298	1.27	No reported
56	Limonene dioxide	232703	8.635	0.23	No reported
57	2,6-Dimethyl-4-nitro-3-phenyl-cyclohexanone	562366	8.664	0.26	No reported
58	Methyl palmitate	8181	8.731	0.46	No reported
59	2,6-Dimethyl-1,3,6-heptatriene	5368331	8.846	0.68	No reported
60	Palmitic acid	985	8.962, 9.020	2.65	Antioxidant, Pesticide
61	Neral	643779	9.077	1.58	Antibacterial, Antispasmodic
62	2-Methyl-6-methylene-1,7-octadien-3-one	93231	9.125	0.75	No reported
63	Bis(3-benzyl-2,4-pentanedionato)palladium(II)	5363840	9.423	1.03	No reported
64	Pentamethylbenzenesulfonyl chloride	590180	9.491, 9.635	6.52	No reported
65	Myrtenal	61130	9.769	3.77	Antimalarial, Antiplasmodial
66	N,N-Dimethyl-2-phenylethen-1-amine	23277871	10.154, 10.183	20.61	No reported
67	Allyl(chloromethyl)dimethylsilane	556526	10.394	7.64	No reported
68	Cyclohexene, 4-(4-ethylcyclohexyl)-1-pentyl-	543386	10.596	1.64	No reported
69	3-Epicycloeucalenol	543796	10.654	1.09	No reported
70	2,5-Furandione, 3-dodecenyl-	5362708	10.750	0.61	No reported
71	1-cinnamyl-3-methylindole-2-carbaldehyde	N/A	10.875	1.35	No reported
72	Glyceryl palmitate	14900	10.962	4.82	No reported
73	2-Methyl-Z,Z-3,13-octadecadienol	5364412	11.414	0.37	No reported
74	Pentadeca-2,3,6,9,12,13-hexaen-8-one, 2,5,5,11,11,14-hexamethyl-	5370200	11.519	0.51	No reported
75	CBMicro_013618	1109374	11.616	1.04	No reported
76	Monoolein	5283468	11.721	2.02	Antioxidant
77	Cyclohexene, 4-(4-ethylcyclohexyl)-1-pentyl-	543386	11.808, 12.596	1.56	No reported
78	Cedrane-8,13-diol	188457	12.654	0.12	No reported
79	26,27-Dinorergosta-5,23-dien-3β-ol	22213488	12.721	0.18	No reported
80	Cholest-4-en-3-one, 14-methyl-	277841	13.279	0.07	No reported
81	(+)-Sesamolin	585998	15.221	0.08	No reported
82	NSC402953	345349	15.308	0.32	No reported
83	Campesterol	173183	15.866	0.12	Antioxidant, Hypocholesterolemic
84	Stigmasta-5,22-dien-3-ol	53870683	16.144	0.06	Antimicrobial, Antioxidant, Antidiabetic
85	Clionasterol	457801	16.923	0.15	Anticancer, Antidaibetic, Antioxidant

**Table 2:** Physicochemical properties of chemical compounds for good oral bioavailability and cell membrane permeability.

No.	Compounds	Lipinski Rules				Lipinski’s Violations	Bioavailability Score	TPSA(Å²)
		MW	HBA	HBD	MLog P			
		<500	<10	≤5	≤4.15	≤1	> 0.1	<140
1	Myrcene	136.23	0	0	3.56	0	0.55	0.00
2	3(5)-[[1,2-Dihydroxy-3-propoxy]methyl]-4-hydroxy-1H-pyrazole-5(3)-carboxamide	231.21	6	5	−2.70	0	0.55	141.69
3	β-Phellandrene	136.23	0	0	3.27	0	0.55	0.00
4	Hex-3-yne	82.14	0	0	3.37	0	0.55	0.00
5	3-Hydroxycyclohexanone	114.14	2	1	0.07	0	0.55	37.30
6	Isopropyl hexanoate	158.24	2	0	2.28	0	0.55	26.30
7	Terpinolene	136.23	0	0	3.27	0	0.55	0.00
8	Vinylcyclooctane	138.25	0	0	4.29	1	0.55	0.00
9	2-Tetradecynoic acid	224.34	2	1	3.58	0	0.85	37.30
10	Citronellal	154.25	1	0	2.59	0	0.55	17.07
11	3-Hydroxy-2,3-dihydromaltol	144.13	4	2	−1.77	0	0.85	66.76
12	Pulegol	154.25	1	1	2.30	0	0.55	20.23
13	Octanoic acid	144.21	2	1	1.96	0	0.85	37.30
14	(E)-4-Undecenal	168.28	1	0	2.88	0	0.55	17.07
15	4-Isopropyl-2-cyclohexenone	138.21	1	0	1.89	0	0.55	17.07
16	Citronellol	156.27	1	1	2.70	0	0.55	20.23
17	(E)--Ocimene	136.23	0	0	3.56	0	0.55	0.00
18	3,7-Dimethylocta-2,6-dien-1-ol	154.25	1	1	2.59	0	0.55	20.23
19	Spiro[4.4]nona-1,3-diene, 1,2-dimethyl-	148.24	0	0	3.56	0	0.55	0.00
20	Piperitone	152.23	1	0	2.20	0	0.55	17.07
21	Nonanoic acid	158.24	2	1	2.28	0	0.85	37.30
22	8,8-Dimethoxy-2,6-dimethyloct-2-ene	200.32	2	0	2.75	0	0.55	18.46
23	p-Isopropylbenzyl formate	178.23	2	0	2.58	0	0.55	26.30
24	Citronellic acid	170.25	2	1	2.47	0	0.85	37.30
25	alpha-Terpinene	136.23	0	0	3.27	0	0.55	0.00
26	2,6-Octadiene, 2,6-dimethyl-	138.25	0	0	3.66	0	0.55	0.00
27	Terpinyl propionate	210.31	2	0	2.92	0	0.55	26.30
28	Geranyl acetate	196.29	2	0	2.95	0	0.55	26.30
29	3-Methylcyclohexene	96.17	0	0	3.33	0	0.55	0.00
30	1,4-Dimethyl-4β-methoxy-2,5-cyclohexadien-1α-ol	154.21	2	1	0.97	0	0.55	29.46
31	2-Propenoic acid, 3-phenyl-, methyl ester	162.19	2	0	2.20	0	0.55	26.30
32	6-Methylenespiro[4.5]decane	150.26	0	0	4.58	1	0.55	0.00
33	beta-Caryophyllene	204.35	0	0	4.63	1	0.55	0.00
34	Bergamotane	208.38	0	0	5.80	1	0.55	0.00
35	3-Methyl-4,7-dioxo-oct-2-enal	168.19	3	0	0.29	0	0.55	51.21
36	2,6-Dimethyl-3,5,7-octatriene-2-ol, Z,Z-	152.23	1	1	2.49	0	0.55	20.23
37	2-Dodecenoic acid	198.30	2	1	3.04	0	0.85	37.30
38	1,6,10-Dodecatrien-3-ol, 3,7,11-trimethyl-	222.37	1	1	3.86	0	0.55	20.23
39	1-Methyldecahydronaphthalene	152.28	0	0	4.72	1	0.55	0.00
40	Cadina-1(10),4-diene	204.35	1	0	4.63	1	0.55	0.00
41	2-(4-Methylcyclohexyl)prop-2-en-1-ol	154.25	1	1	2.30	0	0.55	20.23
42	Tetradec-13-enal	210.36	1	0	3.70	0	0.55	17.07
43	9-Octadecenoic acid	282.46	2	1	4.57	1	0.85	37.30
44	1,2-Di-but-2-enyl-cyclohexane	192.34	0	0	4.37	1	0.55	0.00
45	4,12,12-trimethyl-9-methylene-5-oxatricyclo[8.2.0.04,6]dodecane	220.35	1	0	3.67	0	0.55	12.53
46	3,4-O-Isopropylidene-d-galactose	220.22	6	3	−1.34	0	0.55	88.38
47	2-Hexenoic acid, 6-cyclohexyl-	196.29	2	1	2.65	0	0.85	37.30
48	Heptadec-8-ene	238.45	0	0	6.54	1	0.55	0.00
49	Octane	114.23	0	0	4.20	1	0.55	0.00
50	Myristic acid	228.37	2	1	3.69	0	0.85	37.30
51	D-(-)-Kinic Acid	192.17	6	5	−2.14	0	0.55	118.22
52	Nonadecanoic acid	298.50	2	1	4.91	1	0.85	37.30
53	10-Bromoundecanoic acid	265.19	2	1	3.29	0	0.85	37.30
54	Stearic acid	284.48	2	1	4.67	1	0.85	37.30
55	Cysteamine S-sulfate	157.21	4	2	−1.51	0	0.55	114.07
56	Limonene dioxide	168.23	2	0	1.52	0	0.55	25.06
57	2,6-Dimethyl-4-nitro-3-phenyl-cyclohexanone	247.29	3	0	1.66	0	0.55	62.89
58	Methyl palmitate	270.45	2	0	4.44	1	0.55	26.30
59	2,6-Dimethyl-1,3,6-heptatriene	122.21	0	0	3.26	0	0.55	0.00
60	Palmitic acid	256.42	2	1	4.19	1	0.85	37.30
61	Neral	152.23	1	0	2.49	0	0.55	17.07
62	2-Methyl-6-methylene-1,7-octadien-3-one	150.22	1	0	2.40	0	0.55	17.07
63	Bis(3-benzyl-2,4-pentanedionato)palladium(II)	486.90	4	2	2.69	0	0.85	74.60
64	Pentamethylbenzenesulfonyl chloride	246.75	2	0	3.04	0	0.55	42.52
65	Myrtenal	150.22	1	0	2.20	0	0.55	17.07
66	N,N-Dimethyl-2-phenylethen-1-amine	147.22	0	0	2.40	0	0.55	3.24
67	Allyl(chloromethyl)dimethylsilane	148.71	0	0	2.81	0	0.55	0.00
68	Cyclohexene, 4-(4-ethylcyclohexyl)-1-pentyl-	262.47	0	0	6.61	1	0.55	0.00
69	3-Epicycloeucalenol	426.72	1	1	6.92	1	0.55	20.23
70	2,5-Furandione, 3-dodecenyl-	266.38	3	0	3.53	0	0.55	43.37
71	1-cinnamyl-3-methylindole-2-carbaldehyde	275.34	1	0	3.20	0	0.55	22.00
72	Glyceryl palmitate	330.50	4	2	3.18	0	0.55	66.76
73	2-Methyl-Z,Z-3,13-octadecadienol	280.49	1	1	4.91	1	0.55	20.23
74	Pentadeca-2,3,6,9,12,13-hexaen-8-one, 2,5,5,11,11,14-hexamethyl-	298.46	1	0	4.93	1	0.55	17.07
75	CBMicro_013618	354.40	5	0	1.66	0	0.55	57.90
76	Monoolein	356.54	4	2	3.52	0	0.55	66.76
77	Cyclohexene, 4-(4-ethylcyclohexyl)-1-pentyl-	262.47	0	0	6.61	1	0.55	0.00
78	Cedrane-8,13-diol	238.37	2	2	2.88	0	0.55	40.46
79	26,27-Dinorergosta-5,23-dien-3β-ol	370.61	1	1	6.03	1	0.55	20.23
80	Cholest-4-en-3-one, 14-methyl-	398.66	1	0	6.43	1	0.55	17.07
81	(+)-Sesamolin	370.35	7	0	1.85	0	0.55	64.61
82	NSC402953	386.35	8	0	1.74	0	0.55	73.84
83	Campesterol	400.68	1	1	6.54	1	0.55	20.23
84	Stigmasta-5,22-dien-3-ol	412.69	1	1	6.62	1	0.55	20.23
85	Clionasterol	414.71	1	1	6.73	1	0.55	20.23

**Table 3:** The degree value of target in PPI.

No.	Target	Degree of Value	No.	Target	Degree of Value
1	VEGFA	42	51	ENPP2	9
2	MMP9	28	52	PPARA	8
3	TLR4	23	53	PLA2G2A	8
4	IL2	23	54	NLRP3	8
5	FGF2	23	55	MAOA	8
6	PPARG	22	56	F3	8
7	ESR1	21	57	EDNRA	8
8	PTPRC	19	58	AKR1B1	8
9	AR	19	59	SHBG	7
10	CXCR3	18	60	PTPN6	7
11	MMP2	17	61	PRKCA	7
12	CNR1	17	62	DHFR	7
13	LPAR3	16	63	TYR	6
14	ABCG2	16	64	NR1I3	6
15	NR3C1	15	65	MAOB	6
16	LPAR2	15	66	HMGCR	6
17	LPAR1	15	67	HDAC6	6
18	HDAC1	15	68	ESR2	6
19	ABCB1	14	69	RORC	5
20	S1PR1	14	70	HSD11B1	5
21	CYP2C19	14	71	GSR	5
22	CYP1A2	14	72	FGF1	5
23	CYP19A1	14	73	BCHE	5
24	ALOX5	14	74	RARB	4
25	ABCB1	14	75	HSD11B2	4
26	PLA2G1B	13	76	GRK6	4
27	TNFRSF1A	12	77	GPR35	4
28	PLA2G4A	12	78	GPBAR1	4
29	GABBR2	12	79	DHCR7	4
30	GABBR1	12	80	PTGER2	3
31	CNR2	12	81	PPARD	3
32	PTGES	11	82	PDE4B	3
33	PTGER4	11	83	IL6ST	3
34	NOS2	11	84	HEXB	3
35	MTNR1B	11	85	CES2	3
36	LTB4R	11	86	AKR1B10	3
37	GLI1	11	87	TTR	2
38	EDNRB	11	88	RORA	2
39	TLR9	10	89	KCNA3	2
40	S1PR3	10	90	FABP3	2
41	MMP1	10	91	CTRB1	2
42	HDAC3	10	92	CPA1	2
43	G6PD	10	93	CA2	2
44	CYP17A1	10	94	ASAH1	2
45	ALOX12	10	95	ACP1	2
46	ALDH1A1	10	96	PDE4D	1
47	VDR	9	97	PAM	1
48	TRPV1	9	98	HEXA	1
49	SLC6A4	9	99	GSTK1	1
50	SHH	9			

**Table 4:** Targets in 19 signaling pathways associated with RA.

KEGG ID & Description	Target Genes	False Discovery Rate
hsa04933:AGE-RAGE signaling pathway in diabetic complications	PRKCA,MMP2,VEGFA,F3	0.01010
hsa04926:Relaxin signaling pathway	PRKCA,VEGFA,EDNRB,MMP1,MMP2,MMP9, NOS2	0.00018
hsa04919:Thyroid hormone signaling pathway	PRKCA,ESR1,HDAC1,HDAC3	0.01460
hsa04917:Prolactin signaling pathway	ESR1,ESR2,CYP17A1	0.02200
hsa04915:Estrogen signaling pathway	GABBR1,GABBR2,MMP2,MMP9,ESR1,ESR2	0.00110
hsa04912:GnRH signaling pathway	PRKCA,MMP2,PLA2G4A	0.03510
hsa04660:T cell receptor signaling pathway	IL2,PTPRC,PTPN6	0.03950
hsa04644:Fc epsilon RI signaling pathway	PRKCA,ALOX5,PLA2G4A	0.02070
hsa04370:VEGF signaling pathway	VEGFA,PRKCA,PLA2G4A	0.01690
hsa04151:PI3K-Akt signaling pathway	PRKCA,VEGFA,IL2,TLR4,FGF1,FGF2,LPAR1,LPAR2,LPAR3	0.00110
hsa04072:Phospholipase D signaling pathway	PRKCA,MMP2,LPAR1,LPAR2,LPAR3	0.00660
hsa04071:Sphingolipid signaling pathway	PRKCA,S1PR1,S1PR3,ASAH1,TNFRSF1A	0.00340
hsa04066:HIF-1 signaling pathway	PRKCA,VEGFA,TLR4,NOS2	0.01010
hsa04024:cAMP signaling pathway	PPARA,EDNRA,PDE4B,PDE4D,GABBR1,GABBR2,PTGER2,GLI1	0.00023
hsa04020:Calcium signaling pathway	PRKCA,NOS2,EDNRB,EDNRA	0.03850
hsa04015:Rap1 signaling pathway	PRKCA,VEGFA,FGF1,FGF2,CNR1,LPAR1,LPAR2,LPAR3	0.00025
hsa04014:Ras signaling pathway	PRKCA,VEGFA,FGF1,FGF2,PLA2G2A,PLA2G1B	0.00200
hsa04010:MAPK signaling pathway	PRKCA,VEGFA,FGF1,FGF2,TNFRSF1A,PLA2G4A	0.01810
hsa03320:PPAR signaling pathway	PPARA,PPARD,PPARG,FABP3,MMP1	0.00070

**Table 5:** The degree value of target in STC.

No.	Target	Degree of Value	No.	Target	Degree of Value
1	PRKCA	14	21	TLR4	2
2	VEGFA	8	22	IL2	2
3	MMP2	5	23	PPARD	1
4	PLA2G4A	4	24	PPARG	1
5	FGF2	4	25	FABP3	1
6	FGF1	4	26	ALOX5	1
7	NOS2	3	27	CYP17A1	1
8	ESR1	3	28	S1PR1	1
9	LPAR1	3	29	S1PR3	1
10	LPAR2	3	30	ASAH1	1
11	LPAR3	3	31	GLI1	1
12	PPARA	2	32	PTGER2	1
13	MMP1	2	33	F3	1
14	EDNRB	2	34	CNR1	1
15	MMP9	2	35	HDAC1	1
16	GABBR2	2	36	HDAC3	1
17	GABBR1	2	37	PLA2G2A	1
18	ESR2	2	38	PLA2G1B	1
19	TNFRSF1A	2	39	PTPRC	1
20	EDNRA	2	40	PTPN6	1

**Table 6:** Binding energy of ligands and positive controls on MAPK signaling pathway.

				Grid Box		Hydrogen Bond Interactions	Hydrophobic Interactions
Protein	Ligand	PubChem ID	Binding Energy (kcal/mol)	Center	Dimension	Amino Acid Residue	Amino Acid Residue
FGF1 (PDB ID:3OJ2)	^(★)^ Campesterol	173183	−8.4	x 9.051	x 40	Asp283, Ser252	Arg251,Phe172, Ile257
				y 22.527	y 40		Ser220, Leu258, Ile204
				z −0.061	z 40		Ala260, Gln259, Tyr281
	3,4-O-Isopropylidene-d-galactose	54504880	−6.1	x 9.051	x 40	Arg255, Thr174, Phe172	Asn350, Asn173, Ala349
				y 22.527	y 40	Asn107, Gln348	
				z −0.061	z 40		
Positive control	^(a)^ Suramin sodium	8514	−19.1	x 9.051	x 40	Ser282, Lys27	Arg203, Ile204, Ala260
				y 22.527	y 40		Gln259, Leu258, Tyr281
				z −0.061	z 40		Asn22, Tyr23, Asp283
							Val249, Pro149, Glu250
							His254, Ser252, Ile257
							Phe172, Val222, Ser220
FGF2 (PDB ID:1IIL)	^(★)^ 26,27-Dinorergosta-5,23-dien-3β-ol	22213488	−8.0	x 26.785	x 40	Thr139, Ser137	Glu323, Ser122, Trp123
				y 14.360	y 40		Lys313, Leu312, Ile329
				z −1.182	z 40		Leu327, Tyr328
	Campesterol	173183	−7.9	x 26.785	x 40	Ser137	Thr139, Glu323, Lys313
				y 14.360	y 40		Asp336, Tyr328, Ile329
				z −1.182	z 40		Leu312, Leu327, Ser122
							Trp123, Thr319
	Stigmasta-5,22-dien-3-ol	53870683	−7.8	x 26.785	x 40	Tyr340, Asp336	Ile329, Leu312, Ser122
				y 14.360	y 40		Trp123, Ser137, Thr319
				z −1.182	z 40		Glu323, Asn318, Lys313
							Leu327
	3,4-O-Isopropylidene-d-galactose	54504880	−5.6	x 26.785	x 40	Glu323, Trp123, Ser137	Ser122
				y 14.360	y 40	Thr139, Lys313	
				z −1.182	z 40		
Positive control	^(b)^ NSC172285	299405	−14.7	x 26.785	x 40	Tyr207	Val209, Asp99, Lys119
				y 14.360	y 40		Lys199, Gln200, Glu201
				z −1.182	z 40		
	^(b)^ NSC37204	235612	−9.5	x 26.785	x 40	Thr358, Arg210, Thr121	Val209, Asn265, Lys119
				y 14.360	y 40	Arg118, Glu201	Asp99, Gln200, Trp356
				z −1.182	z 40		
VEGFA (PDB ID: 3V2A)	^(★)^ 3,4-O-Isopropylidene-d-galactose	54504880	−5.3	x 38.009	x 40	Gly312, Ser310	Gly255, Glu44, Ser311
				y −10.962	y 40		Ile256, Asp257, Lys84
				z 12.171	z 40		Pro85
	Glyceryl palmitate	14900	−5.2	x 38.009	x 40	Pro40, Asp276	Arg275, Phe36, Lys286
				y −10.962	y 40		Lys48, Asn253, Ile46
				z 12.171	z 40		
	Monoolein	5283468	−5.1	x 38.009	x 40	Asp276, Pro40	Arg275, Asp34, Asn253
				y −10.962	y 40		Lys48, Phe47, Ile46
				z 12.171	z 40		Phe36, Lys286
	Methyl palmitate	8181	−4.0	x 38.009	x 40	n/a	Pro40, Arg275, Phe36
				y −10.962	y 40		Ile46, Asn253, Lys286
				z 12.171	z 40		Asp276
	Isopropyl hexanoate	16832	−3.9	x 38.009	x 40	n/a	Pro85, Ser310, Gly312
				y −10.962	y 40		Glu44, Ser311, Gly255
				z 12.171	z 40		Gln87, Lys84, Asp257
Positive control	^(c)^ BAW2881	16004702	−7.6	x 38.009	x 40	n/a	Lys286, Asp34, Ser50
				y −10.962	y 40		Asp276, Pro40, Phe36
				z 12.171	z 40		Ile46
TNFRSF1A (PDB ID: 1NCF)	^(★)^ CBMicro_013618	1109374	−6.8	x 21.259	x 40	Lys132, Gln133	Glu109, Tyr106, Gln130
				y 14.648	y 40		Gln133
				z 34.77	z 40		
	2-Propenoic acid, 3-phenyl-, methyl ester	7644	−5.0	x 21.259	x 40	Lys35	Ala62, Glu64, His34
				y 14.648	y 40		Lys35, Glu64
				z 34.77	z 40		
Positive control	^(d)^ Enamine_004209	2340496	−5.3	x 21.259	x 40	Glu109, Cys96, Tyr106	Asn110, Ph112, Val95
				y 14.648	y 40		Gln82, Ser74, Thr94
				z 34.77	z 40		Arg77, Arg132
PLA2G4A (PDB ID: 1BCI)	^(★)^ Stearic acid	5281	−4.5	x −0.058	x 40	Gly33, Lys32	Pro42, Val30, Ile67
				y 0.077	y 40		Val127, Thr31, Gln126
				z 0.285	z 40		
	Methyl palmitate	8181	−3.9	x −0.058	x 40	Lys58	Phe77, Pro54, Thr53
				y 0.077	y 40		Leu79, Tyr16, Glu76
				z 0.285	z 40		Ile78
	Palmitic acid	985	−3.8	x −0.058	x 40	Thr53	Leu79, Phe77, Ile78
				y 0.077	y 40		Tyr16, Glu76, Pro54
				z 0.285	z 40		
	Myristic acid	11005	−3.3	x −0.058	x 40	n/a	Asp55, Pro54, Tyr16
				y 0.077	y 40		Phe77, Ile78, Thr53
				z 0.285	z 40		
Positive control	^(e)^ Berberine	2353	−6.6	x −0.058	x 40	n/a	Arg59, Asp99, Asn95
				y 0.077	y 40		His62, Phe63, Asn64
				z 0.285	z 40		Arg61, Ala94, Tyr45
PRKCA (PDB ID: 3IW4)	^(★)^ Monoolein	5283468	−6.7	x −14.059	x 40	Asn660, Leu393, Asp395	Pro398, Lys478, Glu418
				y 38.224	y 40	Lys396	Pro666, Tyr419, Arg608
				z 32.319	z 40		Val664, Gln402
	Glyceryl palmitate	14900	−6.6	x −14.059	x 40	Asp395, Lys396, Leu393	Leu394, Gln402, Lys478
				y 38.224	y 40	Asn660	Arg608, Pro666, Ile667
				z 32.319	z 40		Val664, Pro398, Pro397
	Stearic acid	5281	−6.3	x −14.059	x 40	Lys396, Leu393	Pro397, Pro398, Lys478
				y 38.224	y 40		Arg608, Ile667, Pro666
				z 32.319	z 40		His665, Val664, Gln402
							Asn660, Leu394
	Nonadecanoic acid	12591	−6.2	x −14.059	x 40	Leu393, Lys396	Asn660, Pro397, Pro398
				y 38.224	y 40		Lys478, Pro666, Arg608
				z 32.319	z 40		Glu418, Val664, Gln402
							Leu394
	1,6,10-Dodecatrien-3-ol, 3,7,11-trimethyl-	8888	−6.2	x −14.059	x 40	Lys372, Gln408, Gln650	Val410, Thr409, Gly540
				y 38.224	y 40		Ile645, Asp539, Asp503
				z 32.319	z 40		Phe538, Glu543
	2,5-Furandione, 3-dodecenyl-	5362708	−6.1	x −14.059	x 40	Lys396, Asp395	Asn660, Gln402, Pro397
				y 38.224	y 40		Pro398, Glu552, Gln662
				z 32.319	z 40		Val664, Leu394
	2,6-Dimethyl-3,5,7-octatriene-2-ol, Z,Z-	5363692	−5.3	x −14.059	x 40	Gly540	Val410, Ile645, Asp503
				y 38.224	y 40		Pro502, Glu543, Gln650
				z 32.319	z 40		Leu546, Asp542
	2-Dodecenoic acid	5282729	−5.1	x −14.059	x 40	Lys396, Asn660, Leu393	Leu394, Gln662, Glu552
				y 38.224	y 40		Val664, Gln402
				z 32.319	z 40		
	9-Octadecenoic acid	965	−5.0	x −14.059	x 40	Leu393, Lys396	Asn660, Glu552, Gln548
				y 38.224	y 40		His553, Ser549, Gln662
				z 32.319	z 40		Val664, Gln402, Leu394
	Octanoic acid	379	−5.0	x −14.059	x 40	Asn660, Lys396, Gln402	Pro397, Lys478, Pro398
				y 38.224	y 40		Val664, Glu552, Arg608
				z 32.319	z 40		
	Methyl palmitate	8181	−5.0	x −14.059	x 40	n/a	Gln377, Asn647, Asp373
				y 38.224	y 40		Ile648, Asp649, Asn468
				z 32.319	z 40		Lys465, Phe350, Asp467
							Ile376
	Palmitic acid	985	−5.0	x −14.059	x 40	Leu393, Asp395, Lys396	Pro397, Pro398, Val664
				y 38.224	y 40		Glu552, His553, Ser549
				z 32.319	z 40		Gln548,Gln662, Gln402
							Leu394, Asn660
	(E)-4-Undecenal	5283357	−4.8	x −14.059	x 40	n/a	Gln642, Pro536, Ile645
				y 38.224	y 40		Gly540, Val410, Gln650
				z 32.319	z 40		Glu543, Asp542, Asp503
							Leu546
	Myristic acid	11005	−4.8	x −14.059	x 40	Lys396, Gln402	Asp395, Leu393, Leu394
				y 38.224	y 40		Pro398, Val664, Gln662
				z 32.319	z 40		Asn660
	Nonanoic acid	8158	−4.7	x −14.059	x 40	Leu393, Lys396	Leu394, Asn660, Pro397
				y 38.224	y 40		Gln402, Gln662, Val664
				z 32.319	z 40		
	Heptadec-8-ene	520230	−4.6	x −14.059	x 40	n/a	Asn647, Asp424, Met426
				y 38.224	y 40		Gln377,Ile376, Phe350
				z 32.319	z 40		Asp467, Asp373
Positive control	^(f)^ Sphingosine	5280335	−5.5	x −14.059	x 40	Asn660, Gln662, Lys396	Pro397, Gln402, Val664
				y 38.224	y 40		Gln548, Glu552, His553
				z 32.319	z 40		Leu394, Ser549, Asp395

^(★)^ Compound with the greatest affinity on each target; ^(a)^ FGF1 antagonist; ^(b)^ FGF2 antagonist; ^(c)^ VEGFA antagonist; ^(d)^ TNFRSF1A antagonist; ^(e)^ PLA2G4A antagonist; ^(f)^ PRKCA antagonist.

**Table 7:** Binding energy of ligands and positive controls on PPAR signaling pathway.

				Grid Box		Hydrogen Bond Interactions	Hydrophobic Interactions
Protein	Ligand	PubChem ID	Binding Energy(kcal/mol)	Center	Dimension	Amino Acid Residue	Amino Acid Residue
PPARA (PDB ID: 3SP6)	^(★)^ β-Caryophyllene	5281515	−8.6	x 8.006	x 40	n/a	Leu321, Leu331, Gly335
				y −0.459	y 40		Val324, Met220, Tyr334
				z 23.392	z 40		Ala333, Thr279, Asn219
							Thr283
	Cadina-1(10),4-diene	10223	−7.4	x 8.006	x 40	n/a	Met220, Leu331, Val324
				y −0.459	y 40		Thr279, Thr283, Leu321
				z 23.392	z 40		Ile317, Met320
	26,27-Dinorergosta-5,23-dien-3beta-ol	22213488	−7.0	x 8.006	x 40	Lys345	Glu356, Asp353, Pro357
				y −0.459	y 40		Leu443, His440, Glu439
				z 23.392	z 40		Leu436, Lys358, Asp360
	Clionasterol	457801	−6.7	x 8.006	x 40	Lys345	Asp360, Pro357, Glu439
				y −0.459	y 40		His440, Leu443, Asp353
				z 23.392	z 40		Glu356
	Cyclohexene, 4-(4-ethylcyclohexyl)-1-pentyl-	543386	−6.6	x 8.006	x 40	n/a	Met320, Phe218, Met220
				y −0.459	y 40		Thr279, Val332, Ala333
				z 23.392	z 40		Tyr334, Thr283, Asn219
	Spiro[4.4]nona-1,3-diene, 1,2-dimethyl-	570800	−6.5	x 8.006	x 40	n/a	Leu321, Leu331, Val324
				y −0.459	y 40		Met320, Asn219, Thr283
				z 23.392	z 40		Thr279, Met220
	1-Methyldecahydronaphthalene	34193	−6.4	x 8.006	x 40	n/a	Met320, Val324, Met220
				y −0.459	y 40		Asn219, Thr279, Thr283
				z 23.392	z 40		Leu321
	1,3,4,5-Tetrahydroxycyclohexanecarboxylic acid	1064	−6.3	x 8.006	x 40	Ile317, Glu286, Asn219	Met320, Met220, Leu321
				y −0.459	y 40	Thr283	
				z 23.392	z 40		
	Stigmasta-5,22-dien-3-ol	53870683	−6.3	x 8.006	x 40	n/a	Arg465, Glu462, Ser688
				y −0.459	y 40		Val306, Asn303, Thr307
				z 23.392	z 40		Tyr311, Gly390, Pro389
							Lys310, Asp466
	Terpinolene	11463	−6.2	x 8.006	x 40	n/a	Thr279, Tyr334, Val324
				y −0.459	y 40		Met220, Met320, Leu321
				z 23.392	z 40		Thr283, Asn219
	β-Phellandrene	11142	−6.0	x 8.006	x 40	n/a	Leu331, Val324, Leu321
				y −0.459	y 40		Ile317, Thr283, Met320
				z 23.392	z 40		Thr279
	Citronellic acid	10402	−5.9	x 8.006	x 40	Thr283, Glu286, Met220	Met320, Asn219, Tyr334
				y −0.459	y 40		Gly335, Leu321, Val324
				z 23.392	z 40		Ile317
	Stearic acid	5281	−5.7	x 8.006	x 40	n/a	Phe361, Asp432, Leu436
				y −0.459	y 40		Glu439, His440, Leu443
				z 23.392	z 40		Asp353, Gln442, Ile446
							Pro357, Lys358
	Monoolein	5283468	−5.7	x 8.006	x 40	Asn261, Lys257	Leu258, His274, Cys275
				y −0.459	y 40		Ala333, Val255, Cys278
				z 23.392	z 40		
	2,6-Octadiene, 2,6-dimethyl-	5365898	−5.7	x 8.006	x 40	n/a	Met320, Phe218, Leu331
				y −0.459	y 40		Val324, Met220, Leu321
				z 23.392	z 40		
	Citronellol	8842	−5.5	x 8.006	x 40	Thr283	Leu331, Val324, Ile317
				y −0.459	y 40		Leu321, Met320, Thr279
				z 23.392	z 40		
	Myrcene	31253	−5.5	x 8.006	x 40	n/a	Val332, Val324, Ile317
				y −0.459	y 40		Leu321, Met220, Thr283
				z 23.392	z 40		Met320, Leu331
	(E)-β-Ocimene	5281553	−5.5	x 8.006	x 40	n/a	Thr283, Ile317, Met320
				y −0.459	y 40		Tyr334, Val332, Val324
				z 23.392	z 40		Gly335, Leu331, Thr279
							Leu321
	Citronellal	7794	−5.2	x 8.006	x 40	Thr283	Leu321, Met220, Met320
				y −0.459	y 40		Val324, Asn219, Thr279
				z 23.392	z 40		Ile317
	Heptadec-8-ene	520230	−5.2	x 8.006	x 40	n/a	Leu321, Ile317, Thr283
				y −0.459	y 40		Thr279, Val255, Ala333
				z 23.392	z 40		Tyr334, Leu331, Val332
							Val324
	Myristic acid	11005	−5.2	x 8.006	x 40	Tyr334, Ala333,Thr279	Val332, Met220, Met320
				y −0.459	y 40		Ile317, Leu321, Thr283
				z 23.392	z 40		Asn219
	Nonanoic acid	8158	−5.1	x 8.006	x 40	Ala333	Leu331, Leu321, Val332
				y −0.459	y 40		Ile317, Thr283, Met320
				z 23.392	z 40		Val324, Thr279
	10-Bromoundecanoic acid	543401	−5.1	x 8.006	x 40	n/a	Met320, Val324, Leu321
				y −0.459	y 40		Thr279, Leu331, Val332
				z 23.392	z 40		Asn219, Tyr334, Met220
	2-Methyl-Z,Z-3,13-octadecadienol	5364412	−5.0	x 8.006	x 40	n/a	Leu254, Ala333, Cys275
				y −0.459	y 40		Tyr334, Ile317, Met320
				z 23.392	z 40		Thr283, Leu321, Leu331
							Val324, Ala250, Thr279
							Ile241, Val255
	Octanoic acid	379	−5.0	x 8.006	x 40	Asn219, Thr283, Met220	Phe218, Leu321, Val324
				y −0.459	y 40	Glu286	Leu331, Met320
				z 23.392	z 40		
	Nonadecanoic acid	12591	−4.9	x 8.006	x 40	n/a	Asn336, Leu254, Ala333
				y −0.459	y 40		Ala250, Cys275, Val255
				z 23.392	z 40		Tyr334
	3-Hydroxycyclohexanone	439950	−4.9	x 8.006	x 40	Met220	Met320, Phe218, Asn219
				y −0.459	y 40		Glu286
				z 23.392	z 40		
	Palmitic acid	985	−4.9	x 8.006	x 40	n/a	Glu251, Val332, Ile241
				y −0.459	y 40		Ala333, Thr279, Val255
				z 23.392	z 40		Tyr334, Leu258, Cys275
							Ala250, Leu254
	3-Methylcyclohexene	11573	−4.6	x 8.006	x 40	n/a	Met320, Ile317, Leu321
				y −0.459	y 40		Thr279, Thr283
				z 23.392	z 40		
	9-Octadecenoic acid	965	−4.4	x 8.006	x 40	n/a	Glu251, Ala250, Leu254
				y −0.459	y 40		Val255, Ile241, Ala333
				z 23.392	z 40		Asn336, Tyr334, Cys275
	2-Tetradecynoic acid	324386	−4.1	x 8.006	x 40	Thr307	Glu462, Ser688, Gln691
				y −0.459	y 40		Tyr311, Lys310, Asn303
				z 23.392	z 40		Val306
	Methyl palmitate	8181	−3.7	x 8.006	x 40	n/a	Tyr311, Gln691, Pro389
				y −0.459	y 40		Lys310, Thr307, Asn303
				z 23.392	z 40		Val306, Ser688, Glu462
Positive control	^(a)^ Clofibrate	2796	−6.4	x 8.006	x 40	Thr283	Ala333, Tyr334, Asn219
				y −0.459	y 40		Met320, Leu321, Met220
				z 23.392	z 40		Phe218, Val332, Val324
							Thr279
	^(a)^ Gemfibrozil	3463	−6.3	x 8.006	x 40	Tyr468	Tyr464, Lys448, Leu456
				y −0.459	y 40		Arg465, Gln442, Ala441
				z 23.392	z 40		
	^(a)^ Ciprofibrate	2763	−5.4	x 8.006	x 40	Ala333, Thr279	Lys257, Cys278, Tyr334
				y −0.459	y 40		Cys275, Val255, Leu258
				z 23.392	z 40		
	^(a)^ Bezafibrate	39042	−5.8	x 8.006	x 40	Thr307, Ser688	Asn303, Glu462, Val306
				y −0.459	y 40		Leu690, Lys310, Gly390
				z 23.392	z 40		
	^(a)^ Fenofibrate	3339	−5.4	x 8.006	x 40	n/a	Gln435, Ala431, Asp360
				y −0.459	y 40		Pro357, Leu436, Glu439
				z 23.392	z 40		Lys364, Phe361, Asp432
PPARD (PDB ID:5U3Q)	^(★)^ Stigmasta-5,22-dien-3-ol	53870683	−8.6	x 39.265	x 40	n/a	Ala414, Tyr441, Met440
				y −18.736	y 40		Pro362, Gly363, Arg361
				z 119.392	z 40		Gly359, Asp360, Thr411
							Val410
	Clionasterol	457801	−7.3	x 39.265	x 40	Met440	Ala414, Tyr441, Tyr284
				y −18.736	y 40		Pro362, Arg361, Asp360
				z 119.392	z 40		Val410, Thr411
	26,27-Dinorergosta-5,23-dien-3beta-ol	22213488	−7.3	x 39.265	x 40	n/a	Lys188, Glu262, Lys265
				y −18.736	y 40		Ser266, Ser271
				z 119.392	z 40		
	Stearic acid	5281	−6.8	x 39.265	x 40	n/a	Glu288, Tyr284, Asp439
				y −18.736	y 40		Asp360, Val367, Gly359
				z 119.392	z 40		Leu364, Gly363, Arg361
							Pro362, Met440, Thr411
							Tyr441
	1,3,4,5-Tetrahydroxycyclohexanecarboxylic acid	1064	−6.6	x 39.265	x 40	Tyr441, Glu288	Ala414, Thr411, Val410
				y −18.736	y 40		Tyr284, Arg361, Arg407
				z 119.392	z 40		Met440
	Nonadecanoic acid	12591	−5.8	x 39.265	x 40	Asp360	Pro362, Tyr441, Met440
				y −18.736	y 40		Val410, Glu288, Arg407
				z 119.392	z 40		Thr411, Arg361, Tyr284
	Citronellal	7794	−5.7	x 39.265	x 40	n/a	Asn307, Ala306, Thr252
				y −18.736	y 40		Trp228, Arg248, Val305
				z 119.392	z 40		Gln230, Lys229
	Nonanoic acid	8158	−5.7	x 39.265	x 40	Thr256	Glu255, Asn307, Lys229
				y −18.736	y 40		Trp228, Ala306, Thr252
				z 119.392	z 40		Glu259, Asn191
	Citronellic acid	10402	−5.3	x 39.265	x 40	n/a	Arg361, Tyr441, Thr411
				y −18.736	y 40		Met440, Tyr284
				z 119.392	z 40		
	Myristic acid	11005	−5.2	x 39.265	x 40	Tyr441	Ala414, Glu288, Pro362
				y −18.736	y 40		Arg361, Asp439, Tyr284
				z 119.392	z 40		Arg407, Thr411, Met440
							Val410
	Octanoic acid	379	−5.1	x 39.265	x 40	Lys229, Gln230, Arg248	Cys251, Trp228, Val305
				y −18.736	y 40		Ala306, Thr252
				z 119.392	z 40		
	9-Octadecenoic acid	965	−5.0	x 39.265	x 40	n/a	Met440, Thr411, Tyr441
				y −18.736	y 40		Pro362, Tyr284, Arg361
				z 119.392	z 40		Val410
	3-Hydroxycyclohexanone	439950	−5.0	x 39.265	x 40	Arg407, Thr411	Val410, Met440, Tyr441
				y −18.736	y 40		
				z 119.392	z 40		
	Citronellol	8842	−4.8	x 39.265	x 40	Arg361	Asp439, Met440, Tyr441
				y −18.736	y 40		Tyr284, Val410
				z 119.392	z 40		
	Palmitic acid	985	−4.6	x 39.265	x 40	n/a	Tyr441, Pro362, Arg361
				y −18.736	y 40		Val410, Tyr284, Glu288
				z 119.392	z 40		Met440, Thr411, Ala414
							Arg407
	10-Bromoundecanoic acid	543401	−4.6	x 39.265	x 40	Arg361, Tyr284	Tyr441, Met440, Pro362
				y −18.736	y 40		Thr411, Glu288
				z 119.392	z 40		
	Methyl palmitate	8181	−4.5	x 39.265	x 40	n/a	Thr411, Tyr441, Pro362
				y −18.736	y 40		Met440, Arg361, Tyr284
				z 119.392	z 40		Glu288
Positive control							
	^(b)^ Cardarine	9803963	−8.5	x 39.265	x 40	Ser271, Ser272	Lys265, Glu262, Ser266
				y −18.736	y 40		Lys265, Ser271, Pro268
				z 119.392	z 40		Ser269
PPARG (PDB ID: 3E00)	^(★)^ NSC402953	345349	−8.2	x 2.075	x 40	Lys336	Asn335, Glu203, Arg234
				y 31.910	y 40		Asp380, Ala231, Lys230
				z 18.503	z 40		Glu378, Asn375, Met334
	26,27-Dinorergosta-5,23-dien-3beta-ol	22213488	−8.0	x 2.075	x 40	Asn375	Val372, Asn335, Val205
				y 31.910	y 40		Val163, Glu207, Glu208
				z 18.503	z 40		
	Stigmasta-5,22-dien-3-ol	53870683	−7.8	x 2.075	x 40	Asn375	Arg202, Glu203, Lys336
				y 31.910	y 40		Val163, Arg164, Gln206
				z 18.503	z 40		Lys165, Glu208, Glu207
							Val372, Asn335
	Clionasterol	457801	−7.7	x 2.075	x 40	Asn375	Asn335, Val372, Lys336
				y 31.910	y 40		Val163, Arg164, Glu208
				z 18.503	z 40		Asp166, Glu207, Arg202
							Glu203
	Terpinyl propionate	62328	−6.6	x 2.075	x 40	Glu343	Leu340, Ile341, Leu333
				y 31.910	y 40		Leu228, Met329, Arg288
				z 18.503	z 40		
	Nonadecanoic acid	12591	−5.9	x 2.075	x 40	Glu351	Lys354, Thr168, Tyr189
				y 31.910	y 40		Tyr169, Thr162, Leu167
				z 18.503	z 40		Tyr192, Arg202, Arg350
							Asp337, Lys336, Gln193
	Stearic acid	5281	−5.9	x 2.075	x 40	Ser342, Cys285	Ile341, Phe226, Ile296
				y 31.910	y 40		Glu295, Ala292, Met329
				z 18.503	z 40		Leu333, Leu228, Arg288
	Citronellic acid	10402	−5.2	x 2.075	x 40	n/a	Glu295, Arg288, Ala292
				y 31.910	y 40		Leu228, Leu333, Met329
				z 18.503	z 40		
	Palmitic acid	985	−5.1	x 2.075	x 40	Glu291, Arg288	Glu343, Leu333, Leu228
				y 31.910	y 40		Met229, Ala292, Ile326
				z 18.503	z 40		Glu295
	Myristic acid	11005	−5.1	x 2.075	x 40	Glu343	Arg288, Ser342, Leu333
				y 31.910	y 40		Met329, Leu228, Glu295
				z 18.503	z 40		Pro227, Ala292
	Nonanoic acid	8158	−5.0	x 2.075	x 40	Lys354	Arg350, Glu351, Tyr169
				y 31.910	y 40		Tyr192, Tyr189, Leu167
				z 18.503	z 40		Gln193, Asp337, Lys336
	Citronellal	7794	−4.9	x 2.075	x 40	Gln193	Tyr189, Arg202, Leu167
				y 31.910	y 40		Thr162, Tyr192, Asp337
				z 18.503	z 40		Lys336, Arg350, Lys354
							Glu351
	(E)-4-Undecenal	5283357	−4.9	x 2.075	x 40	Tyr169	Leu167, Tyr192, Lys336
				y 31.910	y 40		Arg350, Glu351, Asp337
				z 18.503	z 40		Gln193, Lys354, Tyr189
	Octanoic acid	379	−4.7	x 2.075	x 40	Arg202, Leu167	Asp337, Thr168, Tyr192
				y 31.910	y 40		Tyr169, Gln193, Tyr189
				z 18.503	z 40		Glu351, Lys336
	9-Octadecenoic acid	965	−4.1	x 2.075	x 40	Arg164, Glu208	Glu207, Glu203, Asp166
				y 31.910	y 40		Lys336, Arg202, Val372
				z 18.503	z 40		Asn375, Val163
	Methyl palmitate	8181	−3.8	x 2.075	x 40	Glu291, Arg288	Glu343, Leu333, Leu330
				y 31.910	y 40		Leu228, Met329, Ala292
				z 18.503	z 40		Ile326, Glu295
Positive control	^(c)^ Pioglitazone	4829	−7.7	x 2.075	x 40	Arg288	Ile326, Leu333, Met329
				y 31.910	y 40		Ala292, Ile341, Cys285
				z 18.503	z 40		Ser342, Glu343, Glu295
							Leu228
	^(c)^ Rosiglitazone	77999	−7.4	x 2.075	x 40	Tyr169	Glu351, Tyr189, Gln193
				y 31.910	y 40		Thr168, Leu167, Glu369
				z 18.503	z 40		Lys373, Val372, Arg350
							Lys336, Tyr192, Asp337
	^(c)^ Lobeglitazone	9826451	−7.3	x 2.075	x 40	Arg234	Val372, Asn375, Met334
				y 31.910	y 40		Val163, Lys230, Glu203
				z 18.503	z 40		Lys157, Val205, Arg164
							Arg202, Asp166, Lys336
FABP3 (PDB ID: 5HZ9)	^(★)^ Monoolein	5283468	−8.9	x −1.215	x 40	Arg31, Thr57, Phe58	Ala29, Gln32, Phe28
				y 46.730	y 40		Gly27
				z −15.099	z 40		
	Glyceryl palmitate	14900	−8.7	x −1.215	x 40	Arg31	Thr57, Phe58, Ala29
				y 46.730	y 40		Gln32, Phe28, Lys22
				z −15.099	z 40		
	Nonadecanoic acid	12591	−8.3	x −1.215	x 40	Lys22	Ala29, Gln32, Phe28
				y 46.730	y 40		Gly27, Gly25
				z −15.099	z 40		
	Stearic acid	5281	−8.3	x −1.215	x 40	n/a	Gly27, Asp78, Thr122
				y 46.730	y 40		Phe58, Ala29, Phe28
				z −15.099	z 40		Lys22, Lys59, Asp77
							Thr30
	2-Dodecen-1-ylsuccinic anhydride	5362708	−7.8	x −1.215	x 40	Thr30, Gly27	Gly25, Gln32, Ala29
				y 46.730	y 40		Phe28
				z −15.099	z 40		
	Heptadec-8-ene	520230	−7.6	x −1.215	x 40	n/a	Phe28, Met36, Thr57
				y 46.730	y 40		Val33, Ala29, Gln32
				z −15.099	z 40		
	2-Methyl-Z,Z-3,13-octadecadienol	5364412	−7.4	x −1.215	x 40	n/a	Lys22, Gly25, Gly27
				y 46.730	y 40		Gln32, Phe28, Ala29
				z −15.099	z 40		
	2-Tetradecynoic acid	324386	−7.4	x −1.215	x 40	Arg31, Lys22	Phe58, Ala29, Gln32
				y 46.730	y 40		Phe28, Thr57, Lys59
				z −15.099	z 40		
	Methyl palmitate	8181	−7.1	x −1.215	x 40	n/a	Phe28, Gly27, Gly25
				y 46.730	y 40		Gln32, Ala29
				z −15.099	z 40		
	9-Octadecenoic acid	637517	−7.1	x −1.215	x 40	n/a	Ala29, Gly25, Phe28
				y 46.730	y 40		Gly27, Gly25, Gln32
				z −15.099	z 40		
	Myristic acid	11005	−6.9	x −1.215	x 40	Phe58	Ala29, Phe28, Gly27
				y 46.730	y 40		Lys22
				z −15.099	z 40		
	2-Dodecenoic acid	5282729	−6.8	x −1.215	x 40	Arg31	Lys59, Phe58, Thr57
				y 46.730	y 40		Ala29, Gln32, Phe28
				z −15.099	z 40		Lys22
	Citronellic acid	10402	−6.6	x −1.215	x 40	Phe58	Lys22, Met36, Ala29
				y 46.730	y 40		Thr57
				z −15.099	z 40		
	Palmitic acid	985	−6.5	x −1.215	x 40	n/a	Val33, Thr57, Lys22
				y 46.730	y 40		Phe58, Ala29, Gln32
				z −15.099	z 40		
	(E)-4-Undecenal	5283357	−6.5	x −1.215	x 40	n/a	Gln32, Ala29, Gly25
				y 46.730	y 40		Gly27, Phe28
				z −15.099	z 40		
	Isopropyl hexanoate	16832	−6.4	x −1.215	x 40	n/a	Ala29, Phe28, Val33
				y 46.730	y 40		
				z −15.099	z 40		
	Octane	356	−6.1	x −1.215	x 40	n/a	Phe28
				y 46.730	y 40		
				z −15.099	z 40		
	10-Bromoundecanoic acid	543401	−6.1	x −1.215	x 40	n/a	Thr57, Ala29, Gln32
				y 46.730	y 40		Phe28, Lys22, Thr57
				z −15.099	z 40		
	Nonanoic acid	8158	−6.0	x −1.215	x 40	n/a	Gln32, Phe28
				y 46.730	y 40		
				z −15.099	z 40		
	Octanoic acid	379	−5.8	x −1.215	x 40	n/a	Gln32, Val33, Gly25
				y 46.730	y 40		Gly27, Phe28, Ala29
				z −15.099	z 40		
	1,3,4,5-Tetrahydroxycyclohexanecarboxylic acid	1064	−5.6	x −1.215	x 40	Asn307, Ala306, Lys229	Val305, Thr252, Trp228
				y 46.730	y 40		
				z −15.099	z 40		
	Citronellol	8842	−5.4	x −1.215	x 40	n/a	Ala306, Glu255, Asn307
				y 46.730	y 40		Thr252, Trp228, Arg248
				z −15.099	z 40		Gln230, Lys229
	3-Hydroxycyclohexanone	439950	−5.3	x −1.215	x 40	Thr54, Arg107, His94	Thr61, Leu52, Ile63
				y 46.730	y 40		Glu73, Leu105
				z −15.099	z 40		
	Neral	643779	−5.0	x −1.215	x 40	n/a	Arg361, Thr411, Tyr441
				y 46.730	y 40		Met440
				z −15.099	z 40		
	Citronellal	7794	−4.9	x −1.215	x 40	n/a	Thr411, Met440, Pro362
				y 46.730	y 40		Asp360, Val410, Tyr284
				z −15.099	z 40		
MMP1 (PDB ID: 1SU3)	^(★)^ 2-Propenoic acid, 3-phenyl-, methyl ester	7644	−5.0	x 34.394	x 40	Arg399, Tyr411, Tyr397	Asp418, Phe436, Tyr390
				y −44.313	y 40		Phe419, Phe447, Pro449
				z 37.396	z 40		Lys413
	Geranyl acetate	1549026	−4.5	x 34.394	x 40	Tyr411	Arg399, Asp418, Pro449
				y −44.313	y 40		Phe447, Lys452, Phe419
				z 37.396	z 40		Tyr390, Tyr397
	2-Dodecenoic acid	5282729	−4.3	x 34.394	x 40	Tyr397, Tyr411	Lys413, Tyr390, Pro449
				y −44.313	y 40		Phe419, Phe447, Asp418
				z 37.396	z 40		
	Citronellal	7794	−4.0	x 34.394	x 40	Arg399	Phe419, Asp418, Tyr397
				y −44.313	y 40		Phe436, Tyr390, Glu383
				z 37.396	z 40		Pro449
	(E)-4-Undecenal	5283357	−3.6	x 34.394	x 40	n/a	Lys452, Asp418, Pro449
				y −44.313	y 40		Phe436, Phe419, Phe447
				z 37.396	z 40		Tyr390, Tyr397
Positive control	^(d)^ Batimastat	5362422	−6.7	x 34.394	x 40	Thr112, His113, Lys396	Pro146, Glu110, Arg108
				y −44.313	y 40	Thr373	Pro371, Trp398, Pro412
				z 37.396	z 40		Val393
	^(d)^ Ilomastat	132519	−6.5	x 34.394	x 40	His113	Thr145, Ser142, Thr148
				y −44.313	y 40		Leu147, Lys413, His417
				z 37.396	z 40		Met414, Pro412, Gln264
							Pro146

^(★)^ Compound with the greatest affinity on each target; ^(a)^ PPARA agonist; ^(b)^ PPARD agonist; ^(c)^ PPARG agonist; ^(d)^ MMP agonist.

**Table 8:** Toxicological properties of seven key compounds in the MDT.

Parameters	Compound Name						
	Campesterol	26,27-Dinorergosta-5,23-dien-3β-ol	CBMicro_013618	Monoolein	β-Caryophyllene	Stigmasta-5,22-dien-3-ol	NSC0402953
Ames toxicity	NAT	NAT	NAT	NAT	NAT	NAT	NAT
Carcinogens	NC	NC	NC	NC	NC	NC	NC
Acute oral toxicity	I	I	III	IV	III	I	III
Rat acute toxicity	2.8078	2.8078	2.5735	1.0526	1.4345	2.6561	1.9796

AT: Ames toxic; NAT: Non-ames toxic; NC: Non-carcinogenic; category I means (50mg/kg≤ LD50); category II means (50mg/kg > LD50 < 500mg/kg); category III means (500mg/kg > LD50 < 5000mg/kg); category IV means (LD50 > 5000mg/kg).

## Data Availability

All data generated or analyzed during this study are included in this published article (and its Supplementary Materials).

## Supplementary Materials

The following are available online at https://www.mdpi.com/article/10.3390/biology10080703/s1, Table S1: The list of 470, 629, and 215 targets from SEA, STP, and overlapping target proteins between SEA and STP, respectively, Table S2: The list of 3377 target proteins associated with RA, Table S3: The number of final 101 target proteins of ZPFs on RA.

## Abbreviations

AGE	Advanced Glycation End-product;
AMPK	AMP-activated protein kinase;
cAMP	Cyclic AMP;
DMARDs	Disease-Modifying Arthritis Drugs;
FLSs	Fibroblast-Like Synoviocytes;
GC-MS	Gas Chromatography Mass Spectrum;
GnRH	Gonadotropin releasing hormone;
HIF-1	Hypoxia Inducible Factor-1;
KEGG	Kyoto Encyclopedia of Genes and Genomes;
LPS	Lipopolysaccharide;
MAPK	Mitogen Activated Protein Kinase;
MDT	Molecular Docking Test;
NF-κB	Nuclear Factor Kappa B;
NSAIDs	Non-Steroidal Anti-Inflammatories Drugs;
OMIM	Online Mendelian Inheritance in Man;
PI3K-Akt	Phosphoinositide 3-kinase—Akt;
PLD	Phospholipase D;
PLD1	Phospholipase D1;
PPAR	Peroxisome Proliferator Activated Receptor;
PPARA	Peroxisome Proliferator Activated Receptor Alpha;
PPARD	Peroxisome Proliferator Activated Receptor Delta;
PPARG	Peroxisome Proliferator Activated Receptor Gamma;
PPI	Protein-protein interaction;
PRKCA	Protein Kinase C Alpha;
RA	Rheumatoid Arthritis;
RAGE	The Receptor for Advanced Glycation End Products;
Rap1	Ras-associated protein-1;
SEA	Similarity Ensemble Approach;
SMILES	Simplified Molecular Input Line Entry System;
STP	SwissTargetPrediction;
VEGF	Vascular Endothelial Growth Factor;
VEGFA	Vascular Endothelial Growth Factor A;
ZP	Zanthoxylum piperitum;
ZPFs	Zanthoxylum piperitum fruits
