# Supplementary material for: Uncovering Mechanisms of Zanthoxylum piperitum Fruits for the Alleviation of Rheumatoid Arthritis Based on Network Pharmacology
Source: Biology (Basel). 2021 Jul 23;10(8):703. doi: 10.3390/biology10080703 (PMC8389290; doi:10.3390/biology10080703)
Supplement: Supplementary file 1 [file biology-10-00703-s001.zip › Supplementary Table S3.pdf]

101 targets against RA

PPARA

CNR2

AR

CYP19A1

ESR1

ESR2

SHBG

HSD11B1

G6PD

GABBR1

VDR

GPBAR1

AKR1B10

FABP3

PPARD

HSD11B2

HMGCR

PTGER2

CA2
CA1
PAM
CTRB1
MAOA
MAOB
RARB
TRPV1
ALDH1A1
ABCG2
PPARG
MTNR1B
HDAC6
HDAC1
MMP1
TYR
SLC6A4
SLC6A2
BCHE

RORA
PTPN6
CYP2C19
NR3C1
AKR1B1
GSTK1
PTGES
S1PR3
CNR1
F3
ASAH1
CYP17A1
GRK6
MMP9
MMP2
NR1I3
RORC
KCNA3
PTPRC

PDE4D
PLA2G1B
ACP1
LTB4R
NOS2
CES2
PLA2G2A
NLRP3
ALOX5
GSR
TLR4
CA3
PRKCA
TTR
CYP1A2
EDNRA
IL6ST
GABBR2
GLI1

PTGER4
ALOX12
PDE4B
VEGFA
FGF1
FGF2
HEXA
HEXB
CXCR3
PLA2G4A
SHH
GPR35
DHFR
CPA1
ABCB1
S1PR1
TLR9
IL2
HDAC3

TNFRSF1A
ENPP2
LPAR3
LPAR2
LPAR1
EDNRB
DHCR7
