# Supplementary material for: Uncovering Mechanisms of Zanthoxylum piperitum Fruits for the Alleviation of Rheumatoid Arthritis Based on Network Pharmacology
Source: Biology (Basel). 2021 Jul 23;10(8):703. doi: 10.3390/biology10080703 (PMC8389290; doi:10.3390/biology10080703)
Supplement: Supplementary file 1 [file biology-10-00703-s001.zip › Supplementary Table S1.pdf]

470 targets from SEA

SQLE
GGPS1
FNTB
UMPS
TLR2
LPAR4
P2RY10
LPAR6
SLC25A20
GPR174
GPR34
LPAR3
POLM
ACER2
POLH
NDUFAB1
NDUFAF1
NDUFA1
NDUFA2
NDUFA3
NDUFA5
NDUFA6
NDUFA7

NDUFA8
NDUFA9
NDUFA10
NDUFA11
NDUFA12
NDUFA13
NDUFB1
NDUFB2
NDUFB3
NDUFB4
NDUFB5
NDUFB6
NDUFB7
NDUFB8
NDUFB9
NDUFB10
NDUFB11
NDUFC1
NDUFC2
NDUFAF2
NDUFAF3
NDUFAF4
NDUFS1
NDUFS2

NDUFS3
NDUFS4
NDUFS5
NDUFS6
NDUFS7
NDUFS8
NDUFV1
NDUFV2
NDUFV3
MT-ND1
MT-ND2
MT-ND3
MT-ND4L
MT-ND5
MT-ND6
NDUFA4L2
PAM
PLA2G5
CES2
POLK
PPM1A
GNAI1
LPAR2
NDUFA4

PLA2G2C
CES1
MT-ND4
LPAR1
GSTM1
FDPS
FAAH
NOD1
ACP1
ENPP2
GNAI3
ADH1C
EPHX1
GNAO1
FABP3
VEGFA
POLB
NAAA
POLL
DNM1
PLA2G4B
ADH1B
PLA2G2A
PLCG2

SLC22A8
POLA1
SELP
GSTA1
LAP3
HSD17B3
PLA2G10
EPHX2
OXER1
SLC22A6
PRKCE
ADH1A
CYP4F2
ADH7
CDC25A
HMGCR
CDC25C
RARB
HAO1
MSR1
PPARA
GPR84
BBOX1
KDM7A

GABRQ
PHF8
SLC25A20
LPAR6
CERT1
MPEG1
KDM2A
PAOX
SLCO2A1
GSTK1
BHMT
S1PR2
SLC6A11
SMPD2
SELL
LTB4R
SPTLC2
KDM5A
PTGER2
CDC25B
GSR
THRB
KDM4A
S1PR3

TBXAS1
ENPEP
THRA
S1PR4
TBXA2R
PRKCA
FABP4
KDM4E
SPHK1
PTGER4
S1PR5
FOLH1
PTGIR
DAGLA
SLC22A1
PPARG
KDM4C
GABBR2
PTGER3
GABBR1
KDM5C
GPR18
PTGFR
GABRR1

S1PR1
ATG4B
GBA
FFAR4
CPT2
TRPV1
GPR35
PPARD
PLA2G1B
NAALAD2
PAFAH1B2
PDCD4
CNR1
LSS
BTN3A1
FNTA
ADCYAP1R1
RNASEH1
GCGR
TYR
ICMT
CXCL12
NFE2L2
CYP1B1

NR1H4
GPR183
ABCG2
HDAC1
HDAC8
SENP2
AKR1C2
AKR1B10
CHRNA10
CHRNA9
NFKB1
RCOR3
SENP1
AKR1C3
CYP1A1
MAOB
APP
MYOC
MMP1
RELA
HDAC6
HDAC2
MAPT
MMP9

FABP1
PPME1
GLO1
MMP2
TNFRSF1A
HDAC9
GFPT1
ABCB1
TTR
HDAC7
HDAC11
RBBP9
TUBB1
BCHE
HDAC5
CA6
KCNK9
IKBKG
CA5B
RHOA
HDAC4
FABP2
JUN
CA14

TRPM2
ACP3
TOP2A
NLRP1
AKR1B1
CA5A
CA7
ATP6V1B1
CYP27A1
CYP24A1
PARP15
FOS
DPYD
CA3
SENP7
HDAC10
ESR1
CHAT
BACE1
MAOA
ACHE
HDAC3
SLC37A4
GRIK2

KCNK2
AKR1C4
STAT1
NR0B1
SLC12A2
ALOX5
GRIK1
KCNA3
TLR9
F3
MTNR1B
CA9
CYP1A2
NLRP3
HCAR2
CA12
IMPDH2
AKR1C1
IMPDH1
SLC12A5
HTR5A
DHFR
SENP6
KCNQ3

HTR1E
TAAR1
MTNR1A
PARP14
APOBEC3A
CYP2C19
GLI2
GLI1
GPR174
MGLL
SPTLC1
CNR2
TOP1
ALOX12
HEXB
HEXA
GLB1
ADH4
CEL
GLA
LDLR
CBS
SLC22A2
RAD52

GRM6
GRM3
GGH
COL4A3BP
PLA2G4C
PLA2G4A
KCNA1
KCNA4
PAOA
KAT2B
LY96
GBA2
FUT7
IARS1
ASAH1
PGA5
TLR4
APEX1
PTPN13
PTPRC
LPAR5
CTBP2
CPA3
SLC7A5

SLC15A1
CPA1
USP4
DNPEP
USP5
GLS
RNPEP
KCNA2
MB
CACNA1I
P2RX4
IL6ST
AOC2
ANPEP
RPS27
CTRB1
CDC42
LTA4H
FUCA1
CACNA1H
KLK6
NOX1
CAPN2
FFAR1

SLC18A2
CACNA1G
SCN4A
SIGMAR1
KDM4D
CAPNS1
CELA1
P2RX1
SLC1A1
SCN2A
YARS1
SRD5A2
CXCR3
TDP1
G6PC
GRM4
DHCR24
EBP
NPC1L1
ABCC2
ALDH1A1
SPHK2
GAK
CREB1

AXIN2
FSHR
STK17B
KCND3
LYPLA2
GRK6
LYPLA1
ODC1
HCK
GUSB
PDE4B
PI4KB
ERN1
RAD51
PDE4D
CYP17A1
CRYAB
SHBG
SERPINA6
GPBAR1
GC
SLC10A1
FGF2
G6PD

SRD5A1
VDR
NR1H3
CD4
NPC1
SLC10A2
ABCC4
ST3GAL1
ADCY10
ESR2
AR
RORA
SREBF2
ABCB11
SHH
EPHA7
EPHA5
CYP19A1
EPHA8
EPHB3
EPHA4
EPHA1
EPHB1
EPHA6

EPHB2
EPHA2
EPHB6
EPHA3
CA1
GABRD
CA2
NR1H2
SLC22A3
RORC
IL2
CDC45
HSD3B1
HSD11B2
NR3C1
NR3C2
ATIC
EDNRB
EDNRA
PTPRE
GPER1
CYP27B1
FGF1
UGCG

UGT2B7
CYP51A1
SLC6A2
PTPN1
SLC6A4
CHRM2
GLRA1
PTGER1
HSD11B1
PTGES
DHCR7
PTPN6
NR1I3
FDFT1
NOS2

629 targets from STP
PPARA
CNR2
AR
CYP19A1
ESR1
ESR2
SERPINA6
SHBG
HSD17B3
HSD11B1
G6PD
GABBR1
SLC22A6
FNTA
VDR
NPC1L1
NR1H4
GABRA2
POLB
CDC25A
GPBAR1
AKR1B10
FABP4

FABP3
FABP5
PPARD
FFAR1
FABP2
UGT2B7
CHRNA7
HSD11B2
KDM2A
PHF8
KDM5C
HMGCR
PTGER2
PTGFR
GABRB2
GABRG2
CA2
CA1
ADRA2A
ADRA2B
F2
PRSS1
PDE10A
ACHE

CTSH
PAM
ADRA2C
ADRA1A
ADORA1
ADORA2A
ELANE
PTPN1
CA9
FKBP1A
PLA2G6
NISCH
CTRC
CYP11B1
CYP11B2
CTRB1
CCR1
CCR5
CCR8
C1R
KCNK2
TGFBR1
IDO1
TRPM8

MAOA
ADORA2B
ADORA3
MET
FAAH
GABRB3
ALOX15
P2RX7
CRHR1
TNKS2
ALDH3A1
HSP90AB1
PIK3CD
ADRA1D
MAOB
TAAR1
PDGFRA
PSMB1
JAK3
JAK2
TRPA1
PTAFR
PRKCE
PRKCQ

PRKCH
PRKCG
EGFR
PIM1
ICAM1
LIPE
SELE
GABRA5
PDGFRB
PIK3R1
RXRA
RARA
RARG
RARB
SLC16A1
CA12
PTGER1
DHODH
RXRG
AKR1C3
SRD5A2
CCR2
PTGS2
STS

MARS
RXRB
CHRM1
FFAR4
TOP1
HPGD
TRPV1
SRD5A1
ADH1A
ADH1B
ADH7
HLCS
PGR
DRD2
SIGMAR1
DRD4
PTGS1
ALDH1A1
CTSK
CTSL
CTSB
ABCG2
SIRT2
PPARG

MTNR1A
MTNR1B
ADH1C
PSEN2
CTSD
FABP1
HDAC6
HDAC1
MMP13
PARP1
MMP1
CACNA1B
PSMB5
CCND1
MMP8
IKBKB
EPHX2
JAK1
DBF4
HTR2A
PSENEN
NCSTN
APH1A
PSEN1

APH1B
CDC7
CDK4
TYR
DAO
SLC6A4
CHRM2
SLC6A2
BCHE
NR1H3
CA4
SREBF2
RORA
TRPV3
SQLE
HSD17B2
ATP12A
PTPN6
PTPN2
CYP2C19
NR3C1
SAE1
AKR1B1
PLG

GSTK1
CDC45
HAO1
CACNA2D1
PTGDR2
GRM5
PTGES
FDFT1
FNTB
UBA2
S1PR3
GABRA1
GRM1
CTSV
HSP90AA1
GRK2
CNR1
F3
CYP27A1
OPRM1
HSD17B1
NR3C2
NAAA
ASAH1

TGM2
TSPO
DBF4 CDC7
MAPK10
DYRK1A
CA7
NQO2
PIK3CA
DYRK2
DYRK1B
KDR
SLC18A3
AVPR2
CYP24A1
HCRTR2
HCRTR1
CYP17A1
MYLK
GRK6
SCN4A
KCNA5
MMP9
MMP2
MAPK9

PIM3
GABRA3
GABRA6
NR1I2
NR1I3
MAPK3
PTPN11
BACE1
SLC6A3
CES1
CHRNA4
CHRNB2
RORC
TERT
CYP51A1
KCNA3
SCD
KCNH2
HTR7
LTA4H
CXCL8
PTPRC
NOTUM
DUSP3

BCAT2
PTGER3
PTGDR
FADS1
MAPK14
BRD2
GSK3B
CDK2
EPHX1
IMPDH2
ADRA1B
BRD4
HRH3
HRH4
EED
PIM2
BRD3
HTR2C
DRD3
NR4A1
SLC27A1
SUZ12
EZH2
GABRA4

CEL
TNNC1
GSTP1
GSTM2
HSPA1A
PTK2B
DGAT1
TNNT2
TNNI3
PREP
PDE4D
PTPRF
PLA2G1B
ACP1
SLC22A12
CD81
LTB4R
NOS2
CES2
PDPK1
ITGAL
TOP2A
BBOX1
ITGB2

PLA2G2A
TBXA2R
EPAS1
NLRP3
AKR1C2
AKR1C1
ALDH2
METAP1
LSS
ABCC1
VCAM1
PLA2G7
NPY5R
HTT
TBXAS1
TAS2R31
HMOX1
IDH1
PRKDC
PRKCD
QPCT
CYP2A6
FLT3
CXCR2

BCL2L1
F2R
ALOX5
LRRK2
CSF1R
GRM2
BRAF
ABHD6
MGLL
HTR6
SLC8B1
CCNB3
CDK1
TMIGD3
CCNE1
CDK5
HPRT1
ABCC9
KCNJ11
UTS2R
PLAU
AURKA
CDK5R1
TUBB1

RAPGEF4
ERBB2
PGGT1B
CCNB1
CCNB2
GSR
AOC3
HCAR2
CA14
TLR4
KLKB1
CHRNA3
CA3
CA6
CA5B
CA5A
CHRM5
BRD9
CA13
CHRNB4
CHRM3
CHRM4
OPRD1
OPRK1

SCN5A
SCN9A
TYK2
TYMS
MAPK8
KCNE1
ACPP
STK3
PRKCA
CCNE2
PTK2
CYP2C9
LIPA
STK26
CDC25B
TTR
MAP3K8
CSNK2A1
ABAT
MCL1
BCL2
MDM2
MMP12
PDE4A

THRA
THRB
BMP1
CMA1
CTSG
CYP1A2
EDNRA
MMP14
VCP
RPA1
ACE
MMP3
CTNNB1
GCGR
PER2
SLC10A2
PDE2A
TTL
AVPR1A
PABPC1
IL6ST
CYP3A4
PRCP
OXTR

NR1H2
TNK2
CASR
GABBR2
CCR9
BRS3
ICMT
LYPLA2
PRKCB
NOS1
NPY2R
HIF1A
RASGRP1
GCK
OPRL1
GLUL
PYGL
C5AR1
GLI2
GLI1
ROCK2
GAA
HSD3B1
PTGIR

PTGER4
ALOX12
PDE4B
VEGFA
FGF1
FGF2
HPSE
LGALS4
LGALS3
LGALS8
HEXA
HEXB
OGA
CDA
ADA
HTR2B
DRD1
CYP2D6
HTR1B
FUCA1
PYGM
ADK
PNP
TK1

LIG1
KDM4E
STAT3
PTPRG
KDM5A
KDM5B
MIF
KDM4C
SLC16A3
CXCR3
DNM1
RORB
MAPK1
RBP4
PLA2G4A
CYP26B1
CYP26A1
KEAP1
PTGES2
ACLY
FOLH1
AKR1A1
GLRA1
NR0B2

GBA
SHH
LDHA
MSR1
GPR35
PDE7A
KMT5A
FGFR1
ALPL
DHFR
CPA1
ABL1
CCNA1
CCNA2
CPT1A
MPEG1
SLC1A1
GRIN2B
DCK
ABCB1
HNF4A
MPO
KAT2B
TDO2

PIN1
NQO1
HPGDS
HDAC8
AHR
CTSC
SLC18A2
HTR5A
KDM1A
HRH1
ADRB1
HTR1A
PNMT
HTR1D
HTR1E
SLC6A9
SLC9A1
GRIN1
KIF11
ROCK1
CLK4
CLK1
CLK2
CLK3

KDM4A
ACACA
ACACB
QPCTL
AURKB
ADRB3
TACR1
KCNN4
TRPC6
TRPC3
S1PR1
TACR3
TNKS
TLR9
DNMT3A
CTSS
TACR2
G6PC
TRPV4
NMBR
PIK3CB
PIK3CG
LPAR6
LPAR5

IL2
AKT1
PPM1B
PPP1CC
PPP5C
PPP1CA
S1PR5
S1PR4
FASN
GPR119
IKBKE
TBK1
GRM4
CYP1B1
GHSR
HDAC3
HDAC2
PDE3A
PDE3B
TTK
PHOSPHO1
EPHB4
SMO
CACNA2D2

PLAT
KIT
MTOR
TNFRSF1A
CCNC
CDK8
ATM
SCN2A
SCN10A
RPS6KB1
GAK
CSNK2A2
DYRK3
PLEC
TAB1
CCND3
MAP3K7
CCND2
ENPP2
LPAR3
LPAR2
LPAR1
LPAR4
PFKFB3

POLA1
MME
ECE1
VHL
EDNRB
DHCR7

215 overlapping targets between SEA ar
PPARA
CNR2
AR
CYP19A1
ESR1
ESR2
SERPINA6
SHBG
HSD17B3
HSD11B1
G6PD
GABBR1
SLC22A6
FNTA
VDR
NPC1L1
NR1H4
POLB
CDC25A
GPBAR1
AKR1B10
FABP4
FABP3

PPARD
FFAR1
FABP2
UGT2B7
HSD11B2
KDM2A
PHF8
KDM5C
HMGCR
PTGER2
PTGFR
CA2
CA1
ACHE
PAM
PTPN1
CA9
CTRB1
KCNK2
MAOA
FAAH
MAOB
TAAR1
PRKCE

RARB
CA12
PTGER1
AKR1C3
SRD5A2
FFAR4
TOP1
TRPV1
SRD5A1
ADH1A
ADH1B
ADH7
SIGMAR1
ALDH1A1
ABCG2
PPARG
MTNR1A
MTNR1B
ADH1C
FABP1
HDAC6
HDAC1
MMP1
EPHX2

TYR
SLC6A4
CHRM2
SLC6A2
BCHE
NR1H3
SREBF2
RORA
SQLE
PTPN6
CYP2C19
NR3C1
AKR1B1
GSTK1
CDC45
HAO1
PTGES
FDFT1
FNTB
S1PR3
CNR1
F3
CYP27A1
NR3C2

NAAA
ASAH1
CA7
CYP24A1
CYP17A1
GRK6
SCN4A
MMP9
MMP2
NR1I3
BACE1
CES1
RORC
CYP51A1
KCNA3
LTA4H
PTPRC
PTGER3
EPHX1
IMPDH2
CEL
PDE4D
PLA2G1B
ACP1

LTB4R
NOS2
CES2
TOP2A
BBOX1
PLA2G2A
TBXA2R
NLRP3
AKR1C2
AKR1C1
LSS
TBXAS1
ALOX5
MGLL
TUBB1
GSR
HCAR2
CA14
TLR4
CA3
CA6
CA5B
CA5A
PRKCA

CDC25B
TTR
THRA
THRB
CYP1A2
EDNRA
GCGR
SLC10A2
IL6ST
NR1H2
GABBR2
ICMT
LYPLA2
GLI2
GLI1
HSD3B1
PTGIR
PTGER4
ALOX12
PDE4B
VEGFA
FGF1
FGF2
HEXA

HEXB
FUCA1
KDM4E
KDM5A
KDM4C
CXCR3
DNM1
PLA2G4A
FOLH1
GLRA1
GBA
SHH
MSR1
GPR35
DHFR
CPA1
MPEG1
SLC1A1
ABCB1
KAT2B
HDAC8
SLC18A2
HTR5A
HTR1E

KDM4A
S1PR1
TLR9
G6PC
LPAR6
LPAR5
IL2
S1PR5
S1PR4
GRM4
CYP1B1
HDAC3
HDAC2
TNFRSF1A
SCN2A
GAK
ENPP2
LPAR3
LPAR2
LPAR1
LPAR4
POLA1
EDNRB
DHCR7

nd STP
