# Supplementary material for: Uncovering Mechanisms of Zanthoxylum piperitum Fruits for the Alleviation of Rheumatoid Arthritis Based on Network Pharmacology
Source: Biology (Basel). 2021 Jul 23;10(8):703. doi: 10.3390/biology10080703 (PMC8389290; doi:10.3390/biology10080703)
Supplement: Supplementary file 1 [file biology-10-00703-s001.zip › Supplementary Table S2.pdf]

**3,377 targets associated with RA**

PTPN22

SLC22A4

TNF

CRP

IL6ST

CCR6

FCGR2A

PADI4

HLA-DPB1

HLA-DRB1

IL2RA

IL6R

IL10

IRF5

CIITA

STAT4

TNFAIP3

TRAF1

TRAF6
CD28
CD40
PTPN2
TYK2
RUNX1
PTPRC
AGER
IL2RB
NFKBIL1
AFF3
REL
BLK
CDK6
CD244
MMEL1
ANKRD55
GATA3
NFKBIE

PLD4
DNASE1L3
KIF5A
ARID5B
RASGRP1
CSF2
CTLA4
ACAN
RCAN1
AHR
SPRED2
ENO1
ANXA3
HLA-DQA2
IFNG
CCN1
IL1B
IL1RN
IL6

CXCL8
IL18
MIF
MMP2
MPO
MTHFR
NCF2
TNFRSF11B
FOXP3
IL23A
ABCB1
PON1
PTGS2
CCL21
SLC11A1
STAT1
TLR2
VEGFA
CXCR4

CAT
ADIPOQ
CCN2
TNFSF14
DHFR
TNFRSF14
TAGAP
IRAK1
COL2A1
ALOX5
GC
HOXD13
NCF1
TXNDC5
GRK2
FPGS
IGFBP3
FASLG
ATIC

PRKCQ
PTGS1
CCL8
SOD2
GGH
B3GNT2
CP
PLB1
MMP12
ACKR3
PRDM1
CD83
ADORA2A
CLEC12A
IKZF3
FKBP5
CXCL2
HSD11B1
RBPJ

STS
MMP10
BGN
BMP4
SMS
ABCC5
ABCC4
CTSD
DDX6
GRK6
HCLS1
HOXD10
PXK
BCL2A1
BMP6
NR4A3
GDF5
CAV2
ABCC3

GPRC5A
CD2
LHX2
MAB21L2
TXNIP
ABCC2
HAPLN1
GSDME
F2
LY96
GART
RGMB
NR3C1
HOXD11
IRF8
ITGA6
LCN2
MARCKS
RAB8A

PLEK
POU3F1
DDIT4
GIN1
BAIAP2L1
PSG5
NDUFA4L2
PTK2
RAP2A
BDKRB2
CXCL6
TLE3
TMPO
TNFAIP2
ZFP36
TFPI2
CALD1
ST6GALNAC5
MGARP

B3GNT9
RUNX2
SLC25A12
CD3E
CD5
ABCG2
MMP14
ZAP70
DNASE2
HNRNPA2B1
CD226
PADI2
FCRL3
HLA-A
HLA-B
HLA-C
HLA-DQA1
HLA-DQB1
ISG20

TAP2
DPP4
AIRE
MICA
HLA-DMB
ATG5
AIF1
C5
MECP2
NOTCH4
TNIP1
CCR3
HLA-DOA
HLA-DPA1
PTPN11
CFB
SFTPD
TSBP1
CCL27

PSORS1C1
MICB
APOM
RAD51B
BACH2
PRRC2A
DDX39B
LST1
CDSN
RPP14
ICOSLG
PHF19
UBASH3A
PRKCH
GSDMB
BTNL2
COG6
C2
UBE2L3

NELFE
KIAA1109
CASP10
MACIR
SYNGR1
CD247
COL4A1
COL11A2
DAXX
ETFA
RTKN2
HSPA1L
ICAM3
SMTNL2
IL12RB2
FADS1
MBP
EMCN
ATP6V1G2

SUPT20H
PSMB8
PSMB9
WDFY4
RARB
GPSM3
CARD9
TNXB
TPD52
MPIG6B
TMEM187
MYO18B
NTN1
SH3PXD2A
ELMO1
NAT2
ZDHHC20P2
TMPOP1
WAKMAR2

HNRNPA1P2
NONOP2
UBDP1
AP4B1-AS1
CYP4F23P
PSMB8-AS1
MUC22
MSH5-SAPCD1
ATP6V1G2-DDX39B
PPT2-EGFL8
CDH11
TSPAN5
TRIM10
LINC01934
LNCPRESS1
LINC00824
TSBP1-AS1
C3orf67-AS1
C2-AS1

HCG9
LOC102723407
ASB15-AS1
LOC102724971
STAG1
NEBL
LINC02356
LINC02357
LINC02571
ETV7-AS1
LINC01748
AGPAT1
CXCL13
AHSA1
CDC42EP3
EHMT2-AS1
MDC1-AS1
CELF2
TNFSF13B

MTCO2P12
PHTF1
LINC02196
HCP5
PAPOLA
EHMT2
OS9
HTD2
TRIM31
CHI3L1
FAM107A
NRM
IL17F
SMIM40
NLRP3
CSMD3
PHACTR3
RPL3P2
IP6K3

CLIC1
CCR5
ZPBP2
RAVER1
CNTFR
RBM45
FLACC1
TRIM40
MUCL3
SGCZ
ATF6B
CRH
CRK
CRYGD
ASB15
MAPK14
DRAIC
CSNK2B
IL34

CSTF3
IL23R
LINC01104
ZNF595
ERICH1
CYP21A2
DGKQ
MDGA2
ZNF679
CLYBL
DLG2
EGFR
C3orf67
TRIM39-RPP21
EPHB2
AKT1
ESR1
ALB
FCGR3A

FCGR3B
JAZF1
NKAPL
FGF2
GPC5
DKK1
ABCF1
PLCL2
PDS5A
FN1
SIRT1
TNPO3
SDF2L1
IL27
HCG27
WDR27
GABBR1
ASF1A
RNF19A

RCHY1
LY6G6F
MAGI3
POLDIP2
FBXW8
GCH1
GEM
HLA-S
GFRA1
DKK3
IL37
IL17B
HPGDS
GNL1
GPI
LINC00294
CXCR3
HECTD4
TMEM235

HCG22

GSTM1

GSTT1

GUCY1B2

HIF1A

HLA-DOB

HLA-DPB2

HLA-DQB2

HLA-DQB3

HLA-DRA

HLA-DRB4

HLA-DRB9

HLA-G

HMGB1

HSPA4

HSPD1

ICAM1

IFNA1

IFNA13

IGF1
FAS
IL1A
IL2
IL4
IL7
IL9
IL13
IL15
IL17A
ILF3
CXCL10
AR
ITPR3
JAK1
JAK2
JAK3
GALNT18
KDR

KIR3DL1
ZKSCAN4
TMEM179
SFTA2
COL11A2P1
LEP
LGALS3
LINC02649
LINC02656
GATA3-AS1
LMNA
LINC01185
LINC00243
MCCD1
TRIM26BP
MTCO3P1
LTA
MIR146A
MIR155

MIR223
DDR1-DT
HCG18
HCG17
MBL2
MEFV
MAP3K4
MMP1
MMP3
MMP9
MMP13
BCL2L15
MYL8P
MSH5
MSI1
COX2
MTF1
MTX1
LRRC18

NFKB1
NM
NOS3
ACR
NUP88
IL20
IL22
SNHG32
PBX2
CPA4
PDCD1
PDE2A
WNT16
RAB14
CYRIB
SERPINA1
PIK3CA
PIK3CB
PIK3CD

PIK3CG
PLCL1
IL17D
PLG
GDAP1
KRT20
CCHCR1
NECAB2
POU5F1
RSBN1
RBFOX1
PPIAP9
VPS37C
ENOX1
KIF26B
PPP1R10
ACOXL
TCP11L1
CDK5RAP2

ZNF302
MAPK1
MAPK8
PRL
PRTN3
TRIM39
RETN
PSMA4
PRDM10
VARS2
MIR499A
PRR12
DENND1A
ANO8
NCOA5
C6orf47
LY6G5B
LY6G6D
IL21

RARA
BCL2
RELA
ACTB
RIT2
RNF5
RPL37A
RPS18
RPS19
S100A8
SAA1
VPS52
CCL2
CCL5
CCL20
CXCL12
PRDM16
SELE
CLSTN2

NOD2
LINC00452
HLA-DPA2
GORASP1
SKIV2L
WNK1
PCDH15
CASTOR1
SLC6A11
SLC19A1
SOAT1
SPP1
STAT3
SNORA38
BSG
SUOX
SYT1
TAPBP
BTF3P11

TCF19
BTK
TRBV20OR9-2
TEC
TGFB1
TIMP1
TLR3
TLR4
TNFRSF1A
TNFRSF1B
TP53
TRPS1
DCDC2C
TYMS
VARS1
VCAM1
VDR
VIM
VIP

TRIM26
ZNF175
ZSCAN26
DDR1
DDA1
FTO
BAG6
GPANK1
HSD17B8
OR2H2
RNASEH2B
AIMP2
AGBL2
RPP21
ATAT1
SPSB1
ALPK1
CUL5
SLC44A4

VWA7
LY6G6C
LY6G5C
ZNRD1ASP
PRRT1
EGFL8
CDR3
OR5V1
OR12D3
CASP3
UQCC2
DHX16
PGBD1
HAVCR2
AIFM2
CEP89
PPIL4
TNFSF11
TNFRSF11A

SYNGAP1
STK19
NAV2
TRIM15
CCNG2
SOCS3
IL33
RABEP1
IL32
CD14
MS4A1
PPT2
GRAP2
FADS2
GABBR2
CLOCK
CD40LG
CD44
MDC1

CD68
N4BP1
PRORP
RIPOR2
CTIF
SMG7
TNFSF15
EBI3
CDKN2A
CNTRL
CDC42EP1
COMP
PARP1
CX3CR1
ACE
FCGR2B
FOXO3
FOS
HAVCR1

HLA-DMA
IL3
JUN
CX3CL1
THBS1
CCR2
LRPPRC
FSTL1
LINC02605
CSF1
CARD8
IL4R
IL7R
MMP8
WG
PRKN
SOST
TREM1
MAPK3

MYDGF
PTX3
RFC1
CXCR5
ADAM17
TPO
C3
ARHGEF5
SYVN1
TNFRSF6B
IL18R1
TIMELESS
PTGES
SEMA3A
ANP32B
CDAN1
CTSK
CBLL2
ETS1

F2RL1
IFNL1
ANGPT2
CD274
ICOS
CFH
IL12B
KIR2DS1
LPA
MIR21
NFE2L2
NOS2
OSM
P2RX7
SERPINE1
MBL3P
PML
PPARG
S100A9

SAA2
CCL3
TRIM21
TAC1
TLR5
C4B
MUL1
CALR
CASP1
TNFSF13
CD19
CD34
CD69
C4B_2
KLRC4-KLRK1
ADM
HT
DMD
ESR2

KLRK1
TBC1D9
FLT1
FOLR2
IL17RA
GABPA
GCG
GLP1R
ANGPT1
IGHV3-69-1
GZMB
HP
AGBL3
IL16
IRF4
ITGAV
KIR3DL2
LAIR1
LGALS9

LIF
MDK
MYD88
NOTCH1
IL21R
CLEC4A
TLR7
GDE1
TLR9
BANK1
MAP2K7
HAMP
BDNF
CCL7
BRAF
TEK
C4A
SUMO1
BEST1

WNT5A
XRCC1
ZBTB16
CAST
CASP8
TNFRSF25
ADAM15
NR1I2
CD86
ADA
KIR2DS2
CDKN1A
NFAT5
LILRB1
BTG3
CISH
TNFRSF13C
CCR7
CNR2

SLCO6A1
CREB1
CTSL
ADRB2
CYP1A2
OLIG3
DHODH
DNMT1
EGR1
FCGR1A
NLRP1
FOSB
MTOR
ACAD8
B3GAT1
GSTM2
GSTP1
TNC
APOE

IGF1R
LINC01193
IGHG3
IL12A
ITGAM
ITGB1
JUNB
JUND
GSTK1
KLRB1
RPSA
LGALS1
MIR126
MIR22
MIR221
MIR34A
MCL1
MDM2
CXCL9

NFATC1
NR4A2
HSPA14
PLA2G1B
PLAU
PTEN
PTH
CXCL16
RAC1
RARRES2
TNFRSF17
ROS1
S100A4
S100A12
SP1
MAP3K7
TRB
TSPO
TM7SF2

VPS51
VEGFC
DHX40
TSLP
SOCS1
TNFSF10
TNFRSF10A
CFLAR
SQSTM1
CD80
ADAMTS4
GDF15
CD38
HOTAIR
ZGLP1
IL18BP
NAMPT
RABEPK
LANCL1

CDKN2D
NXF1
LOC105379528
KHDRBS1
CXCR6
MALT1
EBNA1BP2
H3P13
H3P28
LRG1
CLU
SERPINA3
CCR4
CR2
CRABP2
CSF1R
CTNNB1
CYBB
DECR1

ATN1
S1PR1
LPAR1
EDNRA
EGF
F3
F9
MMRN1
FOXC1
FLNB
SEC14L2
FPR2
BBC3
GLB1
IGHV3OR16-7
IGHV3-52
TRBV16
TRBV7-9
TRBC1

GPT
GRN
RMC1
IL19
ANXA1
ACACA
HLA-DRB3
HOXD9
APC
HTR2A
ARMH1
IFNB1
IGFBP5
LCE3B
LCE3C
IKBKB
IL5
CXCR2
IL10RA

ITGA1
KIR2DL2
KLRC1
SUMO4
LOC390714
LBP
LOX
MIR10A
MIR145
CD46
MIP
MMP19
PAEP
ANGPTL4
PECAM1
PGF
PRKCA
IL26
PSMD7

MIR451A
CCND1
REG1A
REN
S100A1
S100B
CCL3L1
CXCL5
SELL
SELP
MAP2K4
SFRP1
SHBG
SLC22A5
FSCN1
SOX5
TAP1
TBX5
TPMT

C5AR1
TXN
VWF
STEAP4
EHMT1
CD276
IL1F10
USO1
TP63
CD1C
ARHGEF2
TIMD4
MSC
CD27
ADAM10
DDX39A
SPRY2
SIK1B
KLF2

NOD1

PDPN

TRAF3IP2

HPSE

LILRA3

ADRM1

ADAMTS5

CD160

SLC7A9

POLG2

PRRT2

ACOT7

CTHRC1

H4-16

CCR1

OSCAR

SIK1

CD55

DBP

GADD45A
DNMT3B
DPEP1
E2F2
TYMP
ELAVL2
PRSS55
EPHB1
ERBB2
ERG
EZH2
FCGR1B
FCN1
FHL1
FLG
ACSBG1
FLT3LG
CLEC16A
DICER1

SMUG1
NUP62
SH3BP4
ALOX15
FRZB
AOC1
COPD
MSTN
GDNF
SGSM3
GLS
PYCARD
CXCL1
GTF2H1
GTF2I
GUSB
GZMA
HFE
HLA-DRB5

HMOX1
HOXC6
HSP90AA1
APOB
IDDM8
ACTBL2
H3P44
IGF2
IL1R1
CXCR1
AQP1
TNFRSF9
IRF1
IRS1
ITGA4
ABCC6
ITGAL
ITGB2
KIR2DL1

KLRC2
KLRD1
NPSR1
LRP5
LTB
ARNTL
MIR140
MIR143
MIR20A
MIR23B
MIR27A
MIR17HG
SMAD3
MAP3K5
FOXO4
MPG
MPP1
POTEKP
MIR323A

MIR338
MSRA
MTR
MTRR
MUC1
MYC
GADD45B
NCAM1
NFKBIA
DDR2
OGG1
OLR1
P4HB
PCSK6
PAK3
DCTN4
GP6
TLR8
PDE3A

NCKIPSD
SIRT6
PADI3
SERPINF1
PF4
PFKFB3
PIK3R2
SLCO1C1
ACP5
PPIA
ASPN
SLC52A1
ADI1
MEG3
H4C15
PRKCB
PROC
HTRA1
PSMD12

PTCH1
PTHLH
SEMA6A
SIGIRR
HRH4
RHO
BCR
RPS6KA3
BGLAP
CCL13
CCL18
CXCL11
SDC1
IFIH1
POTEM
ST6GAL1
BMP2
RAPH1
SLC22A1

SLPI
SST
STAT5B
SULT2A1
ABCC8
TACR1
TRA
TFF3
TIE1
TIMP2
TIMP3
ACTG1
TPI1
CRISP2
ACTG2
MUC5B
TNFSF4
TXNRD1
VIL1

VTN
YY1
VTCN1
SCD5
PDCD1LG2
SLC7A5
ADAMTS12
TLR10
CAMP
H4C9
H4C1
H4C4
H4C6
H4C12
H4C11
H4C3
H4C8
H4C2
H4C5

H4C13
H4C14
PRAM1
SPZ1
FCRLA
ORAI1
PIK3R3
ABCC11
BECN1
FADD
CD84
HDAC3
CCK
APLN
PER2
CCRL2
FCGR2C
LPAR2
XPR1

CD6
ASIC3
CD163
KL
FHL5
ENTPD1
BMS1
CDC42
SCO2
MIR760
SH2B3
LINC01672
RPL17-C18orf32
LINC-ROR
ADAM8
CDK2
CALCRL
PRG4
SPRY1

RAMP2
CCL26
WARS2
LINC02210-CRHR1
SEMA4D
CEBPD
KAT5
IVNS1ABP
EBP
CTSC
CFTR
CCR9
CHAT
PSIP1
IRAK3
KLF12
SAAL1
TIRAP
MIR155HG

CHUK
C1QTNF3
C1QTNF6
TADA1
LTB4R
CNR1
PPARGC1B
COX8A
CPB1
CR1
ATF2
CREBBP
CRHR1
CSF3
CSK
CST3
ADRA1A
CTSB
ADRA2B

BTLA
CYP2D6
CYP3A5
CYP17A1
CYP19A1
CYP26A1
DNAJB1P1
NLRP6
DHCR7
AFP
DNASE1
DNTT
DR1
DRD2
JAG1
TSC22D3
DUSP1
EDN1
EFNB1

EIF4EBP1
ELAVL1
ELF3
ELK3
EPAS1
FBL
ETS2
F2R
ALDH1A1
PTK2B
FCER2
FCN2
FGB
FGF1
FGF13
CASC3
COG2
FOXO1
KDM6B

FLT4

BRD4

ALPP

TMEM158

ASPM

GAPDH

ATRNL1

GAS6

HSPB8

GH1

CBLIF

LAMP3

HPLH1

PDLIM3

ABO

CCR10

GPR15

VSTM1

DLL1

TRAV29DV5
GPR42
TRAJ60
TRAC
GPX1
ANPEP
SETD2
GRP
GSK3B
MSH6
SENP1
A1CF
TBX21
SLC40A1
HDAC1
HDGF
ADGRE2
HGF
ANXA6

HIP1

NR4A1

HRAS

HSD11B2

HSPA8

BIRC5

DNAJB1

ICA1

ID1

IFNGR1

IGFBP1

IGHA1

APP

APRT

KLK3

IL1RAP

IL5RA

IL9R

IL10RB

IL11
IDO1
INHBA
INSR
INSRR
AQP9
ITGA2
ITGAE
ITGAX
AREG
KCNMA1
MALAT1
KIR2DL3
KIR2DS5
KLK1
KNG1
ARG1
KPNA1
CCL4L1

LDLR
LGALS2
LGALS3BP
LGALS8
LIG4
LRPAP1
SH2D1A
MIR132
MIR152
MIR17
MIR192
MIR210
MIR214
MIR222
MIR29A
MIR30A
MIR98
SMAD7
MCAM

MET
MFAP1
MFAP4
MKI67
ASIP
MOG
MRC1
MRE11
ZFAS1
MIR346
ASPA
MST1
COX1
MUTYH
MYO9B
ATHS
ATM
NGF
NHS

NME1

NOS1

NOTCH3

NPPA

NT5E

ODC1

OXA1L

G0S2

NOX4

PAK1

PAM

PAX5

ASCC1

ADIPOR1

PCNA

PLEKHO1

PDC

TNFRSF12A

TRPV2

PDE4A
PDE7A
PDGFRA
ENPP2
ADA2
PGR
PITX2
PLA2G4A
TREM2
POU2AF1
ARL15
AHI1
ATG16L1
NAT10
PRELP
CHD7
WDR11
MAP2K1
CCL28

SLC12A9
CEMIP
AICDA
MIR410
KIDINS220
MIB1
PTN
CIP2A
PHRF1
BAK1
RAC2
MOK
RAF1
BCHE
PLAAT4
RB1
UBL5
RBP2
RDX

RNASE2
BCL6
RNF2
IGAN1
RORC
RPL17
SAA3P
SAT1
CCL4
CCL17
CCL19
CCL22
CCL25
SDC2
TINAGL1
SEMA4A
CLEC7A
AGXT2
SLC2A3

SLC6A2
SUMO3
SUMO2
SNCA
SOD1
SOD3
SPARC
SSRP1
SSTR4
STAT5A
STAT6
BRS3
CNTN2
TBCA
GCFC2
TG
TGFA
TGM2
THBD

THOP1
THY1
TIA1
TLR1
TTR
POTEF
TNFRSF4
TYROBP
UQCRFS1
VPREB1
WARS1
WNT3
WNT10B
YWHAZ
CA1
IL1R2
IRX1
ADIPOR2
RHBDF2

ASRGL1
TAM
CAPG
FCRL4
JAM3
LOH19CR1
CASP5
ROPN1L
CASP7
NLRC5
MINDY4
CUL1
IL17RC
SEMA7A
UBASH3B
CAV1
SERPINH1
TNFSF12
TNFRSF10B

IL18RAP
NRP1
CCN6
SPHK1
MBD4
MBD2
PGLYRP1
PSTPIP1
CD1D
PDCD5
INTS4
CD8A
DNER
KLF4
CD22
TNFRSF8
ADAMTS3
TBPL1
CCL4L2

CD48
IKBKE
SDC3
FAM53B
CD70
HDAC9
ADGRE5
PIEZO1
KEAP1
SPATA2
CDKN2B-AS1
MIR708
MIR887
C20orf181
HDAC6
HDAC5
BCL2L11
TRAP
MIR1246

MIR1908
TMED7-TICAM2
MIR762
AAA1
PARP2
DNAJB6
SLC17A4
GAPLINC
LOC100505909
LOC100506023
CNNM3-DT
OCLN
CASP12
H3P43
P2RX5-TAX1BP3
DNM1L
MIR4701
MIR4764
PSC

PTPRU
MIR5571
MIR5100
G3BP1
CHST4
CDK4
CDK7
GDF11
RASGRP2
SLC17A2
MIR6089
MIR6716
MIR6785
CDK2AP2
IRX5
THRIL
CDKN1B
FSTL3
LOC102724334

LOC102725035
LILRB2
DNAJA2
ADAR
CDKN2C
CRISP3
CDKN3
CDR1-AS
CPQ
SPAG11B
YAP1
PRMT5
CFDP1
TMEM147
MERTK
CLEC10A
FST
CREB3
SEMA6B

CEBPB
SEMA3C
MYBBP1A
CIB1
ZNRD2
UPK3B
CERNA3
BATF
CECR
ARPC1A
IFI44
NPC2
LOC106007493
TRIM3
CENPE
POSTN
DCTN6
YME1L1
MAP3K2

MASP2
LOC107987462
CYSLTR1
NEU3
CEACAM8
LYVE1
JTB
SUB1
TMED2
IFI44L
RAB40B
ERVK-32
IL24
LILRA1
LILRB3
LILRA2
PIM2
C5-OT1
TPPP

DNAJB4
RCAN3
CHGA
IL1RAPL1
KAT7
ADAMTS7
TREH
WIF1
CHEK2
CHN2
CHP1
TREX1
B4GALT7
CHRM3
CDCA5
SLC46A1
VSIG4
GLCCI1
CHRNA7

DBA2
UCN3
CIRBP
H3P8
H3P23
H3P19
H3P40
ADCYAP1R1
PRAP1
TPP1
CYP2R1
CLTA
TPH2
CMKLR1
GGT6
PLK3
CNN2
COL1A1
OSCP1

COL1A2
COL4A5
COL9A2
COL12A1
KLF6
ADAD1
ZFP42
CD109
CPA1
CPB2
CLDN7
CPOX
CRABP1
CRHR2
CRKL
ADORA3
MUC17
NRSN1
SIRPA

CSE1L
PIWIL4
CSF2RA
CSF3R
OR2AG1
CSNK1E
PWAR1
B3GNTL1
PTPRVP
CTH
CTRB1
COMMD1
GPBAR1
RMDN2
CTSS
CUX1
CYB5A
CREBRF
CYBA

CYLD
CYP1A1
AMOT
ADRB3
CYP2B6
CYP2C19
CYP2E1
CYP3A4
DCC
DCK
IFNLR1
KANK4
DEFA1
DEFA3
IDO2
AAVS1
NQO1
SARDH
DNAH8

DOCK2
AGT
DSPP
DUSP2
AGTR1
DUSP8
DVL2
DYRK1A
AGTR2
S1PR3
EDNRB
EFNB2
EGR2
AHSG
NLRC3
EIF4E
EIF4G1
ELANE
A2M

ELN
EMD
MARK2
TIGIT
ENG
C9orf72
EPHA1
EPHA3
CLN8
EPO
LVRN
ERBB3
ERCC2
EREG
AKT2
ERV3-1
ESRRB
MECOM
EXT1

ALCAM
F10
ALDH2
FABP5
FAP
FAT1
UNC5B
ALDH1A3
DLEU7
EFEMP1
FCGRT
FGD1
FGF4
FGF7
FGF9
FGFR1
FGFR3
FHL2
VEGFD

IKZF2
NR1I4
PUF60
FOXJ3
SACM1L
CD93
SNW1
P2RX2
FOXJ1
MAST3
ZNF292
MON2
SMG1
ZNF423
TAB2
CIC
FLNA
PHLDB1
FLT3

ARC
SATB2
SYNM
COTL1
HARS2
FOLR1
CABIN1
SNAPIN
DDAH2
LPAR3
FPR1
DDAH1
FAM215A
AMACR
CLEC5A
CD2AP
HSPBP1
PPP1R15A
PLD3

BRD1
ALOX12
ABCA4
ALOX5AP
SPESP1
FTL
FUT4
ZBTB38
EBF3
TAC4
PCSK9
CIZ1
QPCT
PRDX5
PART1
POT1
NCR3
IL4I1
IBTK

LRIG1
PYDC1
LRIT1
KIFBP
GARS1
AMD1
GATA1
FBXO2
GATA2
CLEC4E
GATA4
FGF21
AMD1P2
GCHFR
GCK
KAT2A
SLC17A5
GCY
GDF2

GREM1
AMH
B4GALT1
RNU1-1
GHSR
SMR3A
GJA1
AMPD1
SIGLEC7
LAT
PELP1
DKK2
IL36B
SIGLEC9
GCLC
BMP10
RABGEF1
GLI1
APOBEC3C

GNA12
GNAQ
GNB3
GNS
GPC1
GPLD1
IFNL2
GPR3
ANG
H19
NEAT1
UTS2R
IGHV5-78
IGHV1-69
MIF-AS1
GPER1
CYP4V2
TRBV28
TRBV27

TRBV20-1
TRBV14
TRBV6-7
TRBV3-1
TRBV2
GPR39
TRBC2
TRAV5
GRK5
GRB2
IGKV3D-15
GRIA1
IGKV1D-8
IGKV3-20
IGKV2-29
REM1
LAMTOR2
RGCC
HIPK2

GRIK4
STXBP6
UHRF1
CXCL3
PDIA3
GRPR
GSN
GTS
GYS1
EFEMP2
GZMM
ANXA2
HAGH
HARS1
HAS1
HAS3
HCRTR1
HDC
ANXA4

HEXA
HEXB
ANXA5
KCNIP3
CD209
TAX1BP3
HK1
HK2
HLA-DRB2
HLA-DRB6
HLA-E
HMGCR
HMMR
APAF1
HNRNPC
HOXB2
HOXD4
APCS
HPRT1

HRG

PRMT1

HES1

BIRC2

BIRC3

HSPA1A

HSPA1B

HSPA5

XIAP

HSPB1

HSPB2

NDST1

APOA1

APOA2

IAPP

RSPO2

IDDM7

IDDM13

IFI16

IFI27
IFI35
CYCSP51
IFIT2
IFIT1
IFNGR2
ZNF391
IGFBP4
IGFBP6
IGFBP7
IGH
NMNAT3
IGKC
HLA-P
PADI6
TICAM2
IL2RG
IL11RA
IL12RB1

IL15RA
CYCSP52
INS
INPP5D
IRAK2
IRF3
ISL1
ITGA2B
ITGA5
ITGA9
ITIH1
ITIH4
ITPR2
CD82
KCNA4
MIA3
ENHO
KCNQ1
PICSAR

KIR2DS3
KIR2DS4
KIR3DS1
KIT
KPNA2
ARG2
KRTAP5-9
KRT5
KRT7
KRT8
KRT13
KRT16
THEMIS
RTL1
LAG3
LAIR2
LAMA3
RPS12P4
LBR

LCAT
LCK
LECT2
LIFR
LNPEP
SAPCD1
CRIP3
LOXL2
LERFS
LRP1
LRP2
LRP6
LSP1
LTBR
MIRLET7B
MIR106A
LYZ
MIR125A
MIR137

MIR141
MIR142
MIR147A
MIR150
MIR16-1
MIR18A
MIR182
MIR183
MIR188
MIR193A
MIR196A2
MIR199A1
MIR199A2
MIR19A
MIR19B1
MIR200C
MIR203A
MIR204
MIR212

MIR219A2
MIR23A
MIR26B
MIR27B
MIR29B1
MIR29B2
MIR301A
MIR320A
MIR33A
MIR34B
MIR34C
MIR7-1
MIR7-2
MIR7-3
MIR99B
CAPRIN1
ARRB1
MAA
SMAD1

ARRB2
ASIC1
MAL
MAPT
MARK1
MBNL1
ADAM11
MDM4
MAP3K3
MGAT5
KITLG
ASAH1
MITF
MAP3K11
KMT2A
AFF1
MMP7
MMP15
MMP16

MMP17
MNAT1
MOBP
MOS
MS
CCDC88C
CALHM6
MIR135B
MIR148B
MSD
MSH2
MSH3
TARP
MSMB
PLF
ND1
MTNR1B
MUC2
MUC3A

MUC4
MUC5AC
MX1
MYCN
SERPINC1
ATF3
NBN
NCF4
NDUFA7
NDUFS3
NEDD9
ATOH1
NELL1
NEU1
ATP1A1
NFATC2
NFIA
NFIL3
NFKBIB

NFYA
NNAT
NMT1
NONO
NPAS2
NPPB
NTRK1
NUCB2
OAP
MIR382
OAZ1
ODF1
OPRK1
P2RX1
P2RX3
P2RX4
P2RX5
P2RY1
P2RY2

P2RY11
PEBP1
FURIN
SERPINB2
DEF6
PARD6A
PCBP1
IBD5
EXOSC1
TMED7
NDUFA13
RMDN1
IRAK4
DBR1
ZDHHC2
PCSK1
CLEC1B
RASL12
PCYT1A

ZBTB7A
HOOK1
PDE4D
ISYNA1
NELFCD
ZC3HC1
SCLY
ATP6V1H
PDK1
SF3B6
ENPP1
PDPK1
GHRL
ACP1
PGK1
PGM1
PHEX
PI3
PIGR

PIM1
PIN1
PKD1
PLA2G2A
PLAT
PLAUR
SPA17
PLCG2
PLD1
SERPINF2
PLK1
PLTP
IL20RA
IL20RB
H2BS1
CYCS
POLD1
TERF2IP
SIAE

POMC
PON2
SMOX
ATR
POU2F1
POU2F2
UGT1A10
UGT1A8
UGT1A7
UGT1A6
EGLN1
PPARA
UGT1A4
UGT1A1
PPBP
IL17RD
SAMD9
QPCTL
ZCCHC2

UHRF1BP1
CDKAL1
PPM1A
BANP
MARCHF1
PPP1R1A
TTC38
AKIRIN2
PIWIL2
RMDN3
NADSYN1
THNSL2
KIR2DL5B
IMPACT
MIR448
PRCP
PRF1
SYBU
NLRP2

ZNF334
ATF7IP
DHX32
PAG1
SELENOS
CENPJ
PKN1
PRKCZ
MAPK9
MAPK10
MAPK13
EIF2AK2
ANKH
PROP1
AZGP1
METTL3
PPAN
LTB4R2
PRSS2

MASP1
CYP26B1
PSEN1
SAR1A
SPHK2
PSMB5
ARNTL2
PSMB6
DUSP22
PRDM8
ADAMTS9
PCBP4
PCNP
PNPLA2
CD248
PSMD9
SLURP1
PTBP1
KIR2DL5A

PTGER2
PTGER4
MIR363
ABHD6
MIR432
MIR181D
MIR498
MIR522
MIR506
REXO1
SRGAP1
PTMA
PTMAP4
EPB41L5
MAGEE1
GRHL3
PTPN14
MUC3B
PTPRJ

PTPRK
BAX
RBPJP4
JAM2
RAG1
RAG2
IL22RA1
ACTA2
RAP1A
RARG
HIVEP3
TRPV4
PBOV1
RBP4
OPN1LW
RET
BCL2L1
REV3L
RGS1

RGS12
RHCE
GRK1
BCL3
RMRP
RNASE1
RNASE3
EXOC4
BRD2
SLC5A7
PPCDC
GAS5
ROBO1
RORA
BCS1L
RPS4X
MIR486-1
RRAS
RRM2

RXRA
RYR1
S100A10
S100A11
SAA4
SCD
SRL
CCL1
CCL11
CCL14
CCL23
CCL24
XCL1
SDC4
MRPL17
ERVK-6
ERAP2
SETMAR
STRA6

ROBO3
SFRP2
SFRP4
SFRP5
MMP25
SGCA
DCLRE1C
CIAO3
SGCG
SH3BP2
ITSN1
SHH
ENGASE
P2RY12
IL25
CDCP1
LINC00273
SLAMF1
SLC2A1

SLC3A2
SLC5A5
UCA1
BMP5
SPAG11A
SLC7A4
SLC8A3
SLC22A2
SLIT3
SNAI2
BMX
CHID1
SMO
SMPD1
SIGLEC1
SNAI1
MIR539
SOX4
DST

SPR
BPI
SRC
AKR1D1
SRF
SRP54
SRY
RO60
SSB
ST2
STATH
STIM1
AURKA
TACR2
TACR3
TAGLN
TAL1
TAT
TBX1

TCEA1
PPAN-P2RY11
TBX3
TCF3
MIR421
MIR551B
MIR573
MIR590
MIR613
MIR633
MIR642A
TCF7L2
TCN2
TRAV6
BTN1A1
TRD
TRG
TRGC1
PPP1R11

TDGF1

TDGF1P3

PRDX2

TEF

TEP1

TERF2

TERT

TF

NR2F1

TFDP1

TFF1

TFF2

TGFB2

TGFB3

TGFBI

TGFBR1

TGIF1

THAS

NKX2-1

TNNC1
TNNI3
TP73
TPSAB1
TPT1
NR2C2
HSP90B1
TRAF3
TRAF5
C3AR1
HSP90B2P
CCT3
TRPC1
TRPC5
TRPM2
MIR650
MIR663A
TTN
SFTPA2

TYRO3

TYRP1

UBE2N

UBTF

UCN

UCP2

UGCG

UNG

UTRN

KDM6A

VAV1

VDAC1

VIPR1

TRPV1

WNT1

XBP1

XBP1P1

XRCC4

CA2

CA3
MIR671
MIR766
CACNA1S
PSS
SCG2
MANF
GGCT
AHNAK
ASPSCR1
TSL
DEK
OPLL
BHLHE41
TFEB
NTT
NKAP
SLC52A2
SPAG16

TNFAIP8L2
NLRX1
LRRC31
SHCBP1
CAMKMT
CCDC134
TNIP3
NAA25
TRPM3
CALM1
ZC3H12A
BRD3
WDR26
EFHC2
FER1L4
PDGFD
TET1
PPP1R2C
CEP70

CSRP3
CALM2
FOSL1
FGF23
COL18A1
CALM3
APOL6
AAAS
HMGA2
SLC38A1
GDPD5
LBH
FIP1L1
MAP1LC3B
SLC14A2
MIA
NCOA3
COLQ
EOMES

ARHGAP24
BCL2L12
FAM167A
PARP9
SESN2
ITCH
PLA2G10
TMPRSS13
ZNF644
RECK
CAVIN2
DOT1L
MAP1LC3A
CASR
ZNF469
GPR65
CILP
MAP4K3
PPM1D

TMEM60
DNAJC14
PIR
DCLK3
KHSRP
STC2
PSMG1
HSD17B6
UNC5C
NCOA1
NUMB
MARCO
HYAL2
ACTN1
URI1
RIPK1
TNFSF9
TNFRSF18
CREG1

HESX1
SOCS2
CCN5
CCN4
PROM1
CDK5R1
ALDH1A2
PER3
IER3
ARHGEF7
KRIT1
MTMR3
CCNB1
KYNU
LMLN
RPS6KA4
HSPB3
UCN2
PKD2L1

GADD45GIP1
CCT
CLDN1
MTA1
SLC16A4
SLC16A3
P2RX6
SLC28A2
EBAG9
MFSD4B
OSMR
DEDD
CD4
MTDH
LRAT
NAF1
RPS6KA5
AIMP1
PDLIM7

PIWIL1
CD9
ADGRG1
S1PR2
SRSF11
MMP20
PPIG
LONP1
ZFYVE9
COX5A
CIAO1
CLEC6A
ORMDL3
CYP7B1
AIM2
IL27RA
CHST3
CD36
ATP6V1G1

GTF2IRD1
PRDX6
AKAP12
CD47
NCOR1
ISG15
CD58
CD59
BMS1P20
LPIN2
MARF1
CD63
RIMS2
CD74
CD79A
HDAC4
PCLAF
CDA
GIT2

CDK1
TESPA1
GAB2
NR1I3
HNRNPDL
NDEL1
LACC1
LINC00328
ZMIZ1
SAG
PRKAR1A
ERAP1
RBP3
CALCR
SH2D2A
DGKZ
LRBA
CXADRP1
SPG7

FBN1
GHR
TRIM13
HMGB1P5
CXADR
ANXA11
ITPA
LPP
MIR149
ARR3
HMGB2
HMGN2
HYAL1
RNPC3
MAS1
LGR6
LUM
MGP
VN1R17P

GPR166P
NT5C1A
FZD4
AKNA
HPP1
SOX13
CD5L
PRC1
SPTA1
SNRPD1
TWIST1
TNP1
XRCC6
EMP1
DES
OXER1
TNFRSF13B
GPRC6A
CD3EAP

MRGPRX3
GPR151
MRGPRX4
APOH
BTBD8
GOLGB1
MRGPRX1
GCA
CFHR1
KCNJ15
PLOD2
RBM47
FAM20A
ACP3
ETNK1
PROS1
ZFAND3
HERPUD2
PGM5

KRAS
MAP2
MSN
RERE
ASAP1
MEX3C
MS4A4A
WWOX
PER1
BTG1
TFPI
ACRBP
AOPEP
MIR22HG
SLC22A16
TNFRSF10C
CD8B
B4GALT5
PCYT1B

UBE3C
MTSS1
FCHSD2
MAML2
C2orf88
TNFAIP6
TUBB2A
C8B
UBE2E1
RNF103
DYSF
H2BC8
SH3BGRL2
MAD1L1
RHOBTB1
MYZAP
DDIT3
HBEGF
DUSP4

ZFC3H1
ELF2
APOBEC3A
CTTN
ETV6
DAPK1
CAMSAP1
PLK2
WASF3
FAXDC2
PACSIN2
ADCY7
ANKRD9
CREM
BEND2
CMTM2
RASGEF1B
JMJD1C
R3HDM2

GP1BB
H2AC8
NRG1
HSPA6
TREML1
LILRA5
ITGB5
GNG11
GMPR
TNIK
UHRF1BP1L
AVL9
TTLL5
STAB1
KAT6B
FOSL2
MAFF
TNFAIP8
SIPA1L1

FOXP1
KRT15
TIMMDC1
ATXN2
ZFP36L1
PRR5L
IRF1-AS1
LINC02341
RMI2
PRICKLE2
ZBTB7C
TELO2
COMT
PPP5C
PPT1
SLC6A4
MTG1
OPRM1
FMR1

PRKAA2
GOLPH3
MAOA
GRIN2B
PRKAA1
PRKAB1
SLC6A3
NRXN3
NOL3
PNOC
CES2
MFN2
NPY
OPRD1
KLK4
OPRL1
HTR3B
CNTN3
NPS

SCN9A
TCP1
ZNF77
TP53INP2
TSHR
SNAP25
ABCB6
TRPV3
DRD3
EFNA4
EPHA4
FES
RN7SL263P
PPARGC1A
EMB
DOCK11
C11orf40
MYT1L
IGFALS

L1CAM
LAMC2
MIRLET7D
MAOB
CYTB
PCSK1N
GSR
HTR3A
CPP
APOC1
REEP3
INS-IGF2
C1orf141
ATXN2L
CCDC26
LRRK2
DAG1
LINC01250
CSMD1

NKD1
LURAP1L
GPR35
FNBP1
FAM169B
CACNA1I
LINC00993
CTTNBP2
ADGRL2
IGF2-AS
NRBF2
CRB1
ANKRD30A
ANGPTL5
FUT2
ERAL1
UNC13D
PSMA6
KSR1

C4BPA
EBPL
STXBP2
CARD14
NCR2
FAM177A1
IFN1@
HNF4A
CHIT1
SLC26A2
ZNF395
PRNP
RAB27A
PSMC6
PSMA3
SERPINA2
MME
SOX9
ADAMTS2

CAPS
GZMK
ZBTB22
YWHAE
WNT2
WISP3
WARS
VARS
USP14
URGCP
UBA7
TYR
TSPAN33
TRIM38
TRIM31-AS1
TRIM16
TRGV3
TOP2B
TMPRSS11D

TIPARP
TH
TGM1
TET2
TANK
SYK
ST3GAL5
SSSCA1
SOS1
SLC51B
SLC51A
SLAMF4
SGPL1
SF3B1
SDS
SAA@
S100A6
RUNX1T1
ROCK2

RNU1-4

RBL2

RASGRF1

RARRES3

RAMMET

RAB3GAP1

RA10

RA

PTPRT

PTPN8

PTPN6

PRDX3

PPP3CA

PPARD

PNP

PGAP1

PFF0160c

PERP

PEP

PDLIM4
PDE4B
PAFAH1B1
PADI5
PAD
OCTN1
OA25
NRIP1
NPHS2
NOLC1
NKR2B4
NFKB2
NAIL
MIR7-3HG
MHC2TA
MED27
MCM8
MAPKAPK5
MAPK12

MAPK11
LYP
LY6G6F-LY6G6D
LTC4S
LOC105369230
LOC102723996
LOC100506248
LCP2
KLF15
KDM1A
KCNA3
ITGB7
ITGB3
IL17C
IHH
IFNB2
IBSP
HSF
HSD17B1

HOXD8
HOXD1
HOXA5
HCG23
HCG21
HCG20
HARS
H3F3AP5
GVHDS
GPX2
GFAP
Gene
G6PD
FCAR
FABP3
F13B
F13A1
ESRRA
ERVW-4

ERVW-1
ERVK-9
ERVK-8
ERVK-7
ERVK-25
ERVK-24
ERVK-21
ERVK-19
ERVK-18
ERVK-10
EIF4EBP2
EDA
E2F1
DUSP6
DNAJC2
DDX58
DDX46
DDX19A
CYR61

CTGF
CSRP1
CSIF
COPB2
CHKA
CDK5
CDK10
CCND3
CCNA2
CALB1
CA13
C6orf10
C5orf30
C2TA
BW54
BTG2
BST1
BSF2
BLZF1

BLNK
BDKRB1
BCAR1
ASXL1
ARSD
ALS2CR12
AKR1B10
AKR1B1
ABCA1
BLAUS
SLE
PPRD
SPDA1
JUVAR
WNCHRS
SLEB11
CELIAC6
RPEM
SPENCDI

AIS6
C5ORF30
ILDR2
LAD
A1ATD
DGS
VCFS
IGES
PADI1
PDGFB
TCF12
HEXDC
CACP
CCAL2
IL1RL1
PSORS2
MONA
MAP3K14
MCTO

C2D

MAPKAPK2

FRBRL

MATN1

CHD4

EDSCLL

XLA

HEMA

SSOAOD

WAS

PIK3R1

RPL23A

AD

IBD14

CLL

MAHCF

VAMAS1

MS5

EDSVASC

HD
PD
DA2A
CALJA
SRS5
T1D
FL1
SLEB13
IGAD1
RFH1
AXL
SIGLEC5
TRAT1
TRAPPC2
IRGM
GOLGA8B
CLEC4D
TRNT1
EEF1A1

CCS3, INCLUDED

GP5

TNNI2

BST2

TSPAN8

TNXB1

CYP2C9

FRA2A

SFSWAP

FOXO3A

AIADK

LACH

C8D2

MAN2B2

IMD23

GFI1

FMF

PSORS1

OS1

PSORS13
DIAR5
ADMIO1
HNFJ1
AKU
YAOS
PSORS7
PSORS11
VEGFB
IMD67
HIDS
FCAS2
AILJK
OS2
PRAAS3
PSORS3
MATN3
ALPS2A
GHISID1

CFID
MPLPF
PSORS6
EPCAM
HRH
CINCA
GSD1A
GSD7
PRAAS1
PHOAR1
COA
C3D
C6D
ALPS3
IMD36
ALPS5
EDSPD1
MWS
CVT

DFNA34
CIMAH
IMD57
RLSDF
RLS1
PSORS4
NLRP12
PFKM
SAVI
FERLK
ALPS
ALPS1A
CGD5
SPDA2
PLA2G2F
ADGRB1
HJCYS
CCA
MCKD2

IDD
LDD
HFE3
SEMDX
EDAID1
VEXAS
IPEX
IGHD3
BARTS3
GPHYSD1
GSD1B
GSD1C
GSD1D
HFE1
CILD1
HOMG3
DBQD1
HP1
IBD1

ANFH1
AGM2
C1QD
PHOAR2
EDAID2
IMD68
PFITS
MAHCC
SEDT
APLAID
STLS
AGM7
MMDD
VAIHS
AIFEC
SGMRT2
FLHS
UAQTL1
SEDCJD

MFS
PDB3
JPHT
SVD
STSL1
HFTC1
HFE2A
HFE2
THPM1
CVID8
APRTD
AGM8
IMD53
LPFS3
JRRP
AIEFL
IMD72
PSHB
GPA

SPDA3
DDH2
HSR
T1D7
UOX
C1QC
FFAR2
PTGDR2
SMURF1
PRPS1
MSMO1
COL27A1
RC3H1
CEP250
CANT1
F7
CILP2
PRKCD
C6

RC3H2
IRF2
MOX2
KDM5D
MTATP8
MC5DM2
PTPN12
ARF6
COPA
COPI
CHD3
PSMB4
CYTH2
GOLGA1
LRCH1
